# Host factors of SARS-CoV-2 in infection, pathogenesis, and long-term effects

**DOI:** 10.3389/fcimb.2024.1407261

**Published:** 2024-05-22

**Authors:** Yu Zhang, Shihan Chen, Yan Tian, Xianghui Fu

**Affiliations:** Department of Endocrinology and Metabolism, Department of Biotherapy, State Key Laboratory of Biotherapy and Cancer Center, West China Medical School, West China Hospital and Cancer Center, Sichuan University and Collaborative Innovation Center of Biotherapy, Sichuan, Chengdu, China

**Keywords:** SARS-CoV-2, host factors, pathology, long-term effects, therapeutic targets

## Abstract

SARS-CoV-2 is the causative virus of the devastating COVID-19 pandemic that results in an unparalleled global health and economic crisis. Despite unprecedented scientific efforts and therapeutic interventions, the fight against COVID-19 continues as the rapid emergence of different SARS-CoV-2 variants of concern and the increasing challenge of long COVID-19, raising a vast demand to understand the pathomechanisms of COVID-19 and its long-term sequelae and develop therapeutic strategies beyond the virus *per se*. Notably, in addition to the virus itself, the replication cycle of SARS-CoV-2 and clinical severity of COVID-19 is also governed by host factors. In this review, we therefore comprehensively overview the replication cycle and pathogenesis of SARS-CoV-2 from the perspective of host factors and host-virus interactions. We sequentially outline the pathological implications of molecular interactions between host factors and SARS-CoV-2 in multi-organ and multi-system long COVID-19, and summarize current therapeutic strategies and agents targeting host factors for treating these diseases. This knowledge would be key for the identification of new pathophysiological aspects and mechanisms, and the development of actionable therapeutic targets and strategies for tackling COVID-19 and its sequelae.

## 1 Introduction

Highly pathogenic coronaviruses cause severe respiratory diseases, extra-pulmonary damages, and even death in humans. Over the past two decades, there have been two pandemic outbreaks caused by coronaviruses: severe acute respiratory syndrome (SARS) in 2002 caused by SARS-related coronavirus (SARS-CoV), and Middle East respiratory syndrome (MERS) in 2012 caused by MERS-related coronavirus (MERS-CoV) (Cui et al., 2019). Coronavirus disease 2019 (COVID-19), the ongoing third coronavirus pandemic caused by SARS-CoV-2, has resulted in unprecedented casualties and socioeconomic burden. COVID-19 can manifest in a range of severity, from mild/moderate clinical symptoms to severe or life-threatening diseases (Chen et al., 2020b). The COVID-19 pandemic poses a significant threat not only to elderly individuals with pre-existing health conditions, but also to healthy adults (Gates, 2020). As of March 2024, it has infected over 770 million people worldwide, resulting in approximately seven million fatalities (https://covid19.who.int).

SARS-CoV-2, like all viruses, relies on cellular host factors and pathways to complete its replication cycle successfully. SARS-CoV-2 enters host cells through interactions with cell-surface receptors and proteases. Once inside, the virus engages with intracellular proteins to exploit host mechanisms that facilitate viral replication and evasion of immune responses (Zhou et al., 2023). In this process, SARS-CoV-2 could inevitably induce cellular destruction, disrupt the host immune response, perturb epigenetic regulation, and impair metabolic homeostasis, contributing to the pathogenesis and progression of diseases. Therefore, a better understanding of virus-host interactions is crucial for comprehending SARS-CoV-2 pathogenesis, which would ultimately aid in the identification of novel therapeutic targets and strategies for COVID-19 and related diseases.

The growing number of individuals recovering from COVID-19 has resulted in a heightened focus on long-term effects of this pandemic. Long-COVID refers to a collection of prolonged symptoms that emerge during or after a confirmed or suspected case of COVID-19 (Monje and Iwasaki, 2022). These symptoms encompass various organ systems and commonly include fatigue, breathlessness, headaches, nausea, vomiting, anxiety, depression, skin rash, joint pain, and palpitations (Raman et al., 2022). Moreover, symptoms are merely superficial manifestations. SARS-CoV-2 infection can have lasting and far-reaching effects on the physiological mechanisms and functions of various organs and tissues, not only during the post-acute phase but also persistently in the long term. While long-term organ damage resulting from acute-phase infection is likely responsible for these lasting effects, there may be specific mechanisms following the initial illness that contribute to subsequent pathological changes (Castanares-Zapatero et al., 2022). Therefore, the investigation of host-virus interactions is significant for the prevention and treatment of long-term effects of COVID-19.

This review aims to provide a comprehensive overview of the replication cycle, pathogenesis, and long-term effects of SARS-CoV-2, particularly from the perspective of host factors and host-virus interactions. This knowledge would be key for the identification of new pathophysiological aspects and mechanisms, and the discovery of actionable therapeutic targets and strategies for COVID-19 and related diseases.

## 2 Structure of SARS-CoV-2

**Figure 1:**
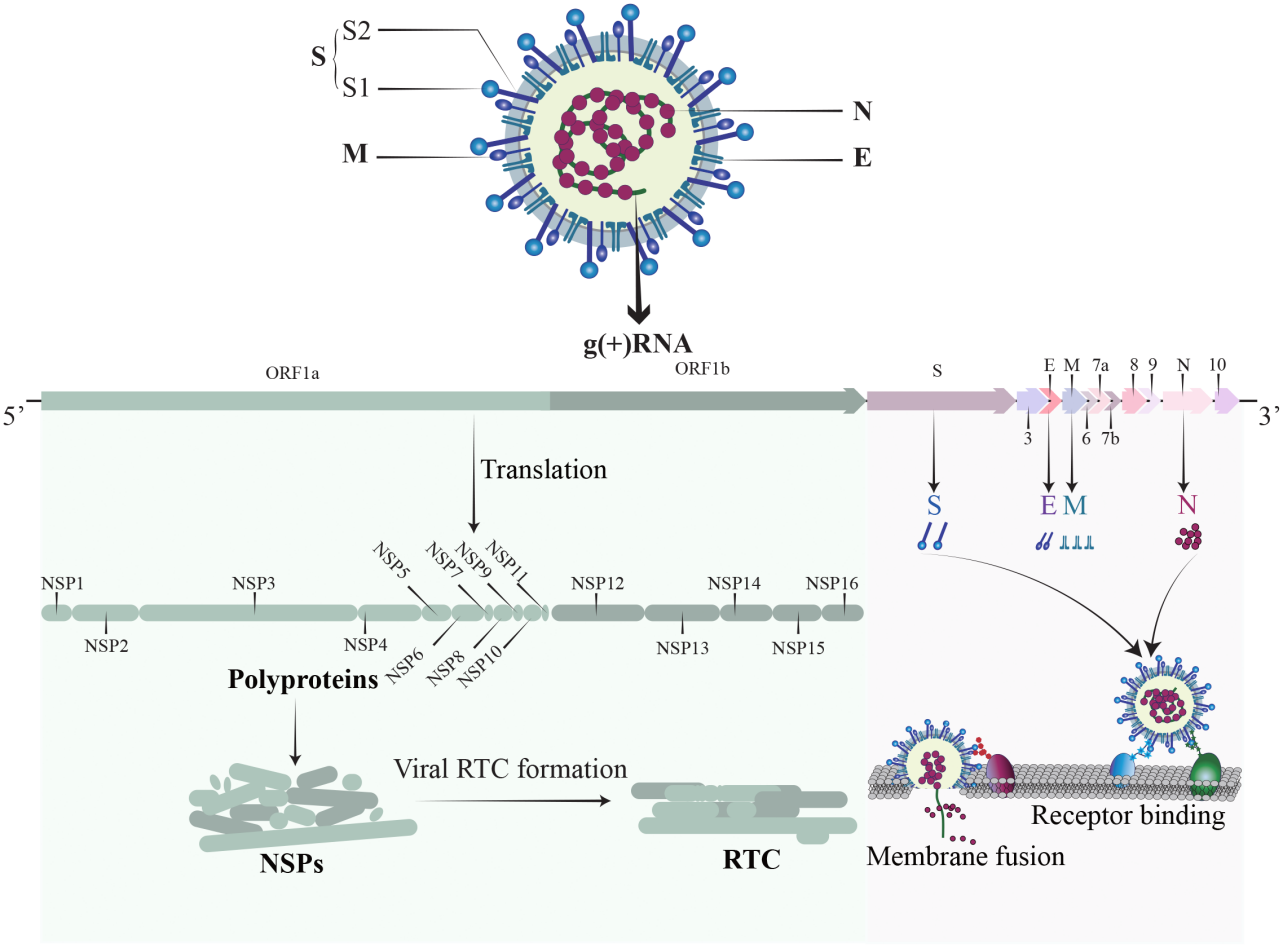
The structure of SARS-CoV-2. SARS-CoV-2 is an enveloped, positive sense single-stranded RNA virus. The RNA genome contains 14 ORFs and 2 untranslated regions. The proteins of SARS-CoV-2 comprise non-structural proteins (NSP1-16), structural proteins (S, M, N, E), and a set of accessory proteins, each serving its own specific function.

SARS-CoV-2, a member of the Coronaviridae family, is an enveloped, positive-sense single-stranded RNA virus (**Figure 1**). The SARS-CoV-2 genome is approximately 30 kb and consists of at least 13 recognized open reading frames (ORFs) and 2 untranslated regions (UTRs) (Malone et al., 2022). The protein component of SARS-CoV-2 is composed of non-structural proteins (NSPs), structural proteins, and accessory proteins. NSPs are crucial for RNA-dependent RNA polymerase (RdRp) holoenzyme formation, replication organelle formation, and viral protein synthesis (Malone et al., 2022; Li et al., 2023a), and play a vital role in immune evasion and abnormal inflammatory response (Zhou et al., 2021). The structural proteins of SARS-CoV-2 include a nucleocapsid protein (N), a spike protein (S), a membrane protein (M), and an envelope protein (E) (Li and Chang, 2023). The N protein encapsidate the viral RNA genome and forms a protective nucleocapsid, shielding the viral RNA from cytoplasmic immune surveillance while facilitating the assembly of nucleoprotein complexes (Lu et al., 2011; Scherer et al., 2022). The S protein is vital for the entry of SARS-CoV-2 (Yu et al., 2023). Following enzymatic cleavage at the furin site, the S protein undergoes division into two subunits: S1 and S2 (Jackson et al., 2022). The S1 subunit forms the spike head and contains the receptor binding domain (RBD) that is primarily responsible for receptors recognition, and the S2 subunit attaches to the viral envelope and forms a stem-like structure that activates membrane fusion (Walls et al., 2020). The M protein is indispensable for virus assembly and contributes to the formation of viral envelope (Zhang et al., 2022b), while the E protein has a role in vesicle transport, budding, and intercellular transmission. The accessory proteins are recognized as crucial virulence factors in various pathogenesis pathways during SARS-CoV-2 infection, primarily contributing to immune evasion (Zandi et al., 2022). In short, the structure of SARS-CoV-2 is relatively simple, yet its nucleic acids and proteins play multiple and vital roles in viral replication and pathogenesis.

## 3 Host factors in SARS-CoV-2 replication cycle

**Figure 2:**
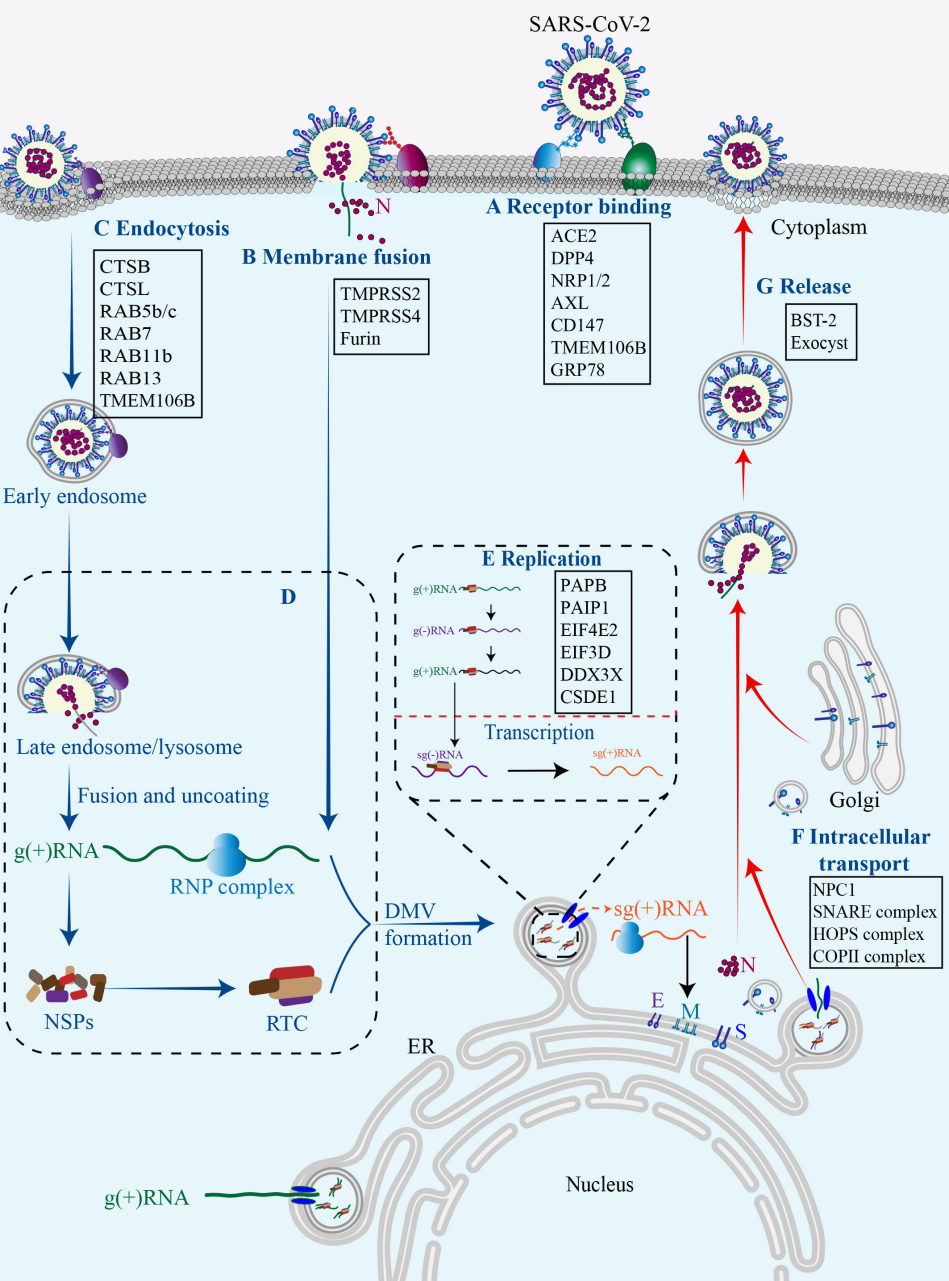
Host factors in SARS-CoV-2 replication cycle.Numerous host factors are required for the completion of SARS-CoV-2 replication cycle. In brief, SARS-CoV-2 attaches to susceptible cells and the S protein binds to cell surface receptors **(A)**. SARS-CoV-2 enters the cell through membrane fusion **(B)** or endocytosis **(C)**. The virus releases its genetic material into the cell, and the viral gRNA initially functions as an mRNA and subsequently undergoes translation to generate NSP1-16, which then form the replication/transcription complex **(D)**. In double-membrane vesicles, the viral gRNA serves as a template for replication and production of both gRNAs and sgRNAs. The latter functions as mRNA to synthesize structural proteins and other accessory proteins **(E)**. The transportation of viral components within the cell occurs through the host membrane system **(F)**. The newly synthesized gRNAs, structural proteins, and accessory proteins assemble into new virions which are subsequently released from infected cells via budding **(G)**.

The replication cycle of SARS-CoV-2 generally consists of the following stages: specific engagement of the S protein with host cell receptors; entry of virus into the host cell through membrane fusion or endocytosis; synthesis of viral RNA and proteins; assembly of progeny virions and their ultimate release (Chen et al., 2023b). Notably, the simple structure of SARS-CoV-2 alone is insufficient for completing its full replication cycle, and a number of host factors dynamically participate in each juncture of this intricate procedure (**Figure 2**) (Rajarshi et al., 2021).

### 3.1 Entry

Thus far, there are two pathways responsible for the entry of SARS-CoV-2 into host cells, namely the membrane fusion pathway and the endocytosis pathway, both of which are initiated with the binding of the viral proteins to cell surface receptors. In both pathways, the S protein serves as an indispensable “promoter”. In the membrane fusion pathway, the S protein is cleaved on cell surface by host proteases, such as type II transmembrane serine protease (TMPRSS2), to assists the fusion of viral envelope with the cytoplasmic membrane (Glowacka et al., 2011). In the absence of cell surface proteases, SARS-CoV-2 may gain entry into host cells through endocytosis pathway. Within the endosome, cathepsin cleavage activates the S protein to initiate fusion between the viral envelope and the endosomal membrane (Ou et al., 2021). Upon cleavage and activation, the S protein undergoes conformational changes that result in the conversion from a fusion-competent state to a membrane-embedded homotrimeric prehairpin, followed by further transformation into a trimer-of-hairpin structure. During these transitions, the S protein exposes the fusion peptide, leading to the formation of fusion pores in the membrane through tight adhesion and semi-fusion, ultimately complete membrane fusion (White et al., 2008).

Cell surface receptors are essential for SARS-CoV-2 infection. Among them, angiotensin-converting enzyme 2 (ACE2) serves as the predominant receptor for SARS-CoV-2 entry, primarily by interacting with the RBD on the S1 subunit through its peptidase domain (Samelson et al., 2022). The binding mode of SARS-CoV-2 and ACE2 closely resembles that of SARS-CoV (Oudit et al., 2023). However, in comparison to SARS-CoV, the binding of SARS-CoV-2 S protein and ACE2 demonstrates more extensive interactions and increased affinity (Evans and Liu, 2021). In addition to ACE2, other cell surface receptors can also interact with the S protein and facilitate virus infection. Dipeptidyl peptidase 4 (DPP4), also known as CD26, is a transmembrane protein and the primary receptor for MERS-CoV, and thus has been considered a potential receptor for SARS-CoV-2. Indeed, DPP4 can interact with S protein RBD through its four residues (Mani et al., 2023). Interestingly, the membrane-anchored DPP4 is a virus receptor, while the soluble form (sCD26) acts as a competitive inhibitor to prevent the virus-DPP4 interaction (Raha et al., 2020). AXL, a tyrosine protein kinase receptor enriched in the lung and trachea, is another receptor for SARS-CoV-2 by interacting with the N-terminal domain (NTD) of the S protein (Fang et al., 2023). The co-localization of the S protein with AXL and endocytosis/vesicle transport markers in infected cells suggests that AXL may boost virus internalization through a clathrin-dependent mechanism (Wang et al., 2021c). The cleavage of the S protein by furin protease generates a binding site for neuropilin-1 (NRP1) and NRP2, indicative of potential SARS-CoV-2 receptors (Cantuti-Castelvetri et al., 2020). In addition, CD147 has been reported to interact with the S protein and facilitate the entry of SARS-CoV-2 through endocytosis (Wang et al., 2020b). Altogether, accumulating data suggest that the ACE2 receptor plays a pivotal role in facilitating SARS-CoV-2 cell entry, while other receptors may synergistically collaborate with ACE2 to enhance viral infectivity. However, the presence of certain receptors in cells lacking ACE2 expression expands the range of SARS-CoV-2 tropism, thereby rendering virtually all tissue cells susceptible to SARS-CoV-2 infection. It is of profound significance for future study to unravel these receptors and their action mechanisms.

Thus far, three cellular proteases are primarily involved in the hydrolytic activation of the S protein. Among them, TMPRSS2 plays an essential role in facilitating the fusion of the SARS-CoV-2 envelope with the host cell plasma membrane by cleaving the S protein at the S2’ sites and exposing the fusion peptide (Shapira et al., 2022). Interestingly, TMPRSS2 has been observed to co-localize with ACE2 in various cell types, including lung type II pneumocytes, ileal absorptive enterocytes, and nasal goblet secretory cells. It not only provides a reasonable explanation for the vulnerability of these cells to SARS-CoV-2, but also highlights the significance of TMPRSS2 as a pivotal host protease in viral invasion (Ziegler et al., 2020). Consistent with this, the absence of co-expression of ACE2 and TMPRSS2 in individual pancreatic β cells diminishes the probability of direct SARS-CoV-2 infection *in vivo* (Coate et al., 2020). Another serine protease called TMPRSS4 can also enhance S protein cleavage and facilitate virus entry. Although TMPRSS4 is less effective than TMPRSS2, its abundance in human intestinal epithelial cells may predominantly contribute to virus infection in the gastrointestinal tract (Zang et al., 2020). Furin protease, renowned for its ability to cleave envelope glycoproteins of influenza and human immunodeficiency virus (HIV) (Follis et al., 2006). can pre-cleave the S protein at the S1/S2 boundary, resulting in S1 and S2 subunits. The pre-cleavage enhances subsequent cleavage by TMPRSS2 and contributes to the high infectivity and tropism of SARS-CoV-2 (Essalmani et al., 2022). Insufficient expression of TMPRSS2 or failure of a virus-ACE2 complex to encounter TMPRSS2 can result in the internalization of SARS-CoV-2 through the clathrin-mediated endosomal/lysosomal pathway. The cleavage of the S2’ site can be conducted by Cathepsin L (CTSL) and CTSB, leading to the initial fusion between the viral envelope and endosomal membrane (Hoffmann et al., 2020; Takeda, 2022).

Although several host proteases are involved in SARS-CoV-2 infection, the activation of the S protein is predominantly promoted by TMPRSS2, and the fusion process occurring at the cell surface represents the most efficient mechanism for viral entry (Jackson et al., 2022). It implies that targeting the process of membrane fusion could be more effective in preventing viral infection compared to endocytosis (Ou et al., 2021). In other words, inhibitors focusing on the endocytosis pathway demonstrate limited efficacy in restricting SARS-CoV-2 infection, while a combined approach involving a TMPRSS2 inhibitor alongside an endocytosis pathway inhibitor holds potential advantages.

### 3.2 Replication

Upon entering the cytoplasm, the SARS-CoV-2 genome initiates its replication program. The viral genomic RNA (gRNA) initially serves as messenger RNAs (mRNAs) for translation. ORF1a and 1b encode the polyproteins PP1a and PP1ab respectively, which are subsequently cleaved by viral proteases into 16 distinct NSPs. NSP1 is responsible for halting the translation of host mRNAs, while the remaining NSPs mainly form replication-transcription complexes (RTCs) to facilitate viral RNA synthesis (Lou and Rao, 2022). The replication of the viral genome occurs within double-membrane vesicles (DMVs) that are perinuclear membrane structures derived from the endoplasmic reticulum (ER) and induced by SARS-CoV-2 infection (Snijder et al., 2020). Within DMVs, the gRNA acts as a template for synthesizing negative strand RNA intermediates, which in turn serve as templates for synthesizing new positive strands. The SARS-CoV-2 genome produces a total of 10 distinct (+) RNAs, comprising one full-length gRNA and nine sub-genomic RNAs (sgRNAs) (Wyler et al., 2021). Transmembrane pores facilitate the transportation of the newly formed viral RNAs from the DMV to the endoplasmic reticulum-Golgi intermediate compartment (ERGIC) for subsequent translation (Wolff et al., 2020). The translation of sgRNAs in the cytoplasm produce SARS-CoV-2 structural proteins (S, E, M, and N) as well as accessory proteins (3a, 3c, 6, 7a, 7b, 8, and 9b) (Lee et al., 2021).

The hijack of host machinery by SARS-CoV-2 is crucial for viral RNA replication and protein synthesis (Fung and Liu, 2019), yet it significantly impairs the normal gene expression of the host. NSP1 is a critical virulence factor and contributes to the host translational shutoff at multiple stages (Frolov et al., 2023), NSP1 can interact with the heterodimer NXF1-NXT1, a receptor for host mRNA export on the nuclear pore complex, and inhibit the cytoplasmic translocation of host mRNAs (Zhang et al., 2021a). NSP1 may also disrupt the translation of host mRNAs by inserting its C-terminal domain into the mRNA channels of the ribosomal 40s subunit (Schubert et al., 2020). Moreover, NSP1 is able to promote the degradation of host mRNAs by cleaving their 5’-UTR, thereby accelerating cellular mRNA decay mediated by the 5’-3’ exoribonuclease 1 (Lou and Rao, 2022; Tardivat et al., 2023). NSP3, also known as the SARS-unique domain (SUD), enhances the affinity between polyA-binding protein (PAPB, an essential translation factor) and PABP-interacting protein 1 (PAIP1), and thus promotes protein synthesis of SARS-CoV-2 rather than the host (Lei et al., 2021b). SARS-CoV-2 also causes DNA damage and disrupts the DNA damage response, resulting in genomic instability. Specifically, ORF6 and NSP13 cause the degradation of DNA damage response kinase CHK1 through proteasomal and autophagic mechanisms respectively, leading to DNA damage. The N protein may interfere with damage-induced long non-coding RNAs (lncRNAs) to impair 53BP1 focal recruitment and DNA repair (Gioia et al., 2023). Recently, a comprehensive SARS-CoV-2-human protein-protein interactome reveals a physical interaction between ORF3a and zinc finger protein 579 (ZNF579), an uncharacterized human protein likely functioning as a transcription factor, suggesting a potential impact on ZNF579-modulating transcriptome (Zhou et al., 2023).

To facilitate gene replication and expression, the viral RNA recruits specific host RNA-binding proteins (RBPs) to form ribonucleoprotein (RNP) complexes. Recently, a deep learning tool was employed to identify SARS-CoV-2-interacting RBPs and revealed numerous candidates, including heterogeneous nuclear ribonucleoprotein A1 (hnRNPA1), hnRNPK, hnRNPU, U2 small nuclear RNA auxiliary factor 2 (U2AF2), interleukin enhancer binding factor 3 (ILF3), TIA1 cytotoxic granule associated RNA binding protein (TIA1), insulin like growth factor 2 mRNA binding protein 1 (IGF2BP1), and staphylococcal nuclease and tudor domain containing 1 (SND1) (Sun et al., 2021b). Indeed, depletion of TIA1, SND1 and IGF2BP1 significantly reduced the production of SARS-CoV-2 RNA in infected cells (Sun et al., 2021b). It is worth mentioning that ILF3 is an important protein required for interleukin-2-expressing T cells, and can also form complexes with other proteins, dsRNAs, small non-coding RNAs (ncRNAs), and mRNAs, to widely regulate gene expression, thereby participating in the occurrence and development of a variety of tumors (Jiang et al., 2021; Zhang et al., 2023). However, its role and mechanism in COVID-19 remain unclear.

Besides, eukaryotic translation initiation factor 4E type 2 (EIF4E2), EIF3D, DEAD-box helicase 3 X-linked (DDX3X), and cold shock domain containing E1 (CSDE1) have been identified as pro-viral RBPs (Hoffmann et al., 2021; Lee et al., 2021). In contrast, zinc finger CCCH-type containing, antiviral 1 (ZC3HAV1), tripartite motif containing 25 (TRIM25), poly (ADP-ribose) polymerase family member 12 (PARP12), shiftless antiviral inhibitor of ribosomal frameshifting (SHFL), and RNA binding motif protein 24 (RBM24) are probably anti-viral RBPs (Lee et al., 2021; Yao et al., 2023). For instance, ZC3HAV1, activated by TRIM25 through ubiquitination, exerts the anti-viral effect by promoting RNA degradation and inhibiting translation (Zheng et al., 2017), and SHFL may obstruct ribosomal coding during ORF1 translation (Schmidt et al., 2021). These findings suggest that the status of virus replication is partially dependent on the equilibrium and dynamic responses of pro- and anti-virus RBPs.

### 3.3 Transport and release

After synthesis, the viral genome and proteins are transported to ERGIC for assembly, leading to the formation of complete progeny virions that are then released from the cell surface through exocytosis (Ravi et al., 2022). It is increasing known that this transportation is intricately linked to the host membrane system. Intriguingly, approximately 40% of the host proteins involved in the interaction with SARS-CoV-2 are associated with endomembrane compartments or vesicle trafficking pathways (Gordon et al., 2020). It is reasonable to speculate that the regulation of intracellular transport of viral components by host factors is likely to exert a significant impact on the viral replication cycle.

In the endocytosis pathway, SARS-CoV-2 is transported to early endosomes in a RAS-associated protein 5 (RAB5)-dependent manner through the interaction between the S protein and RAB5b/c (Chen et al., 2021). The S protein can also interact with RAB11b and RAB13, which are associated with early endosomes and regulate the trafficking of receptors and other ligands to the membrane. In late endosomes, the S protein may interact with late endosomal proteins, including RAB7a, RAB7b, and RAB7l1, to potentially facilitate lysosomal maturation (Tiwari et al., 2021). While in the cytoplasm, the M protein and the ORF7b possess the capability to interact with several components of the soluble NSF attachment protein receptor (SNARE) complex, an essential complex for all membrane fusion process, such as syntaxin 6 (STX6) and STX10. Additionally, the ORF3a interacts with the homotypic fusion and protein sorting (HOPS) complex, which regulates membrane vesicle transport through mechanisms involving membrane fusion. Furthermore, NSP13 may interact with the Golgi complex (Gordon et al., 2020), while the ORF6 protein binds to components of the coatmer protein II (COPII) complex that is crucial for vesicle formation and substance transportation from the endoplasmic reticulum to the Golgi apparatus (Chen et al., 2021). In short, the interactions between virus and host proteins maximize their intracellular transportations. Among them, lysosomal transmembrane protein 106B (TMEM106B) is a prominent representative (Wang et al., 2021b). TMEM106B plays important roles in lysosomal transport, acidification, and lysosomal enzyme expression, and it is thus reasonable that it can regulate viral infection through these actions (Luningschror et al., 2020). Moreover, it has recently reported that TMEM106B may function as an alternative receptor for SARS-CoV-2 entry into ACE2-negative cells and facilitate the formation of spike-mediated syncytia (Baggen et al., 2023), further expanding its implication in SARS-CoV-2 pathogenesis. The exocyst, identified through CRISPR screens, is an octameric protein complex that facilitates the tethering of secretory vesicles to the plasma membrane. Consequently, this complex may enhance viral particle trafficking during entry or egress and regulate surface expression of viral entry factors (Mei and Guo, 2018; Wang et al., 2021b). It has been proved that SARS-CoV-2 infection efficiency was regulated by EXOC2, one of key subunits of the exocyst complex (Yi et al., 2022).

There are also host proteins that play a role in the release phase of the virus. Bone marrow stromal antigen 2 (BST-2), also known as tetherin, is found in the plasma membrane, trans-Golgi network, and circulating endosomes, and can inhibit virus release (Tiwari et al., 2019). Mechanistically, BST-2 interacts with the S protein and thus forms a bridge between the budding virions and the plasma membrane, tethering the new virions to the plasma membrane to inhibit their release (Tiwari et al., 2019, Tiwari et al, 2021). However, this anti-viral activity can be impeded by ORF7a that directly binds to BST-2 and hinders its glycosylation process (Mann et al., 2023).

During SARS-CoV-2 infection, viral proteins extensively remodel host cell endomembrane through interactions with host factors to facilitate various stages of the viral cycle, Including the formation of DMVs for viral replication and the modulation of the lysosome pathways for virus release. The endomembrane system is of great potential to serve as a therapeutic target against SARS-CoV-2, its emerging variants, and even novel coronaviruses.

## 4 Host genetic factors in SARS-CoV-2 susceptibility and COVID-19 severity

It is well established that the severity and fatality risk of COVID-19 are intricately linked to the viral load of SARS-CoV-2 (Fajnzylber et al., 2020; El Zein et al., 2021; Tarafder and Khan, 2023), which in turn may be influenced by host gene polymorphism (Roy-Vallejo et al., 2023). Consequently, recent research highlights that the heterogeneity observed in the clinical manifestation of acute SARS-CoV-2 infection may be attributed, at least partially, to human genetic variation (Redin et al., 2022). This notion is highlighted by ACE2, the pivotal receptor for SARS-CoV-2. Two ACE2 variants are associated with increased SARS-CoV-2 susceptibility: the minor A allele in the rs2106806 variant and the minor T allele in the rs6629110 variant, with the later leading to a substantial increase in ACE2 expression (Martinez-Sanz et al., 2021). An *in vivo* study in hamsters suggests that higher ACE2 expression was associated with a higher viral load and more intense post-acute sequelae (Delval et al., 2023). Genetic polymorphism of ACE2 affect not only the expression of ACE2, but also the affinity between ACE2 and the S protein (Chan et al., 2020). The interaction between the S protein and ACE2 can be increased by certain single nucleotide polymorphisms (SNPs), such as rs73635825 and rs1244687367, but decreased by others, including rs1348114695 and rs1192192618 (Calcagnile et al., 2021; Lanjanian et al., 2021; Suryamohan et al., 2021).

Polymorphisms in other host genes, such as TMPRSS2, DPP4, interferon-lambda-3, tolloid like-1, discoidin domain receptor 1 and HLA-DRB1*08, may also contribute to the susceptibility and severity of COVID-19 (Irham et al., 2020; Senapati et al., 2020; Agwa et al., 2021; Amoroso et al., 2021). A meta-analysis revealed a significant correlation between TMPRSS2 rs12329760 C-allele and an elevated risk of developing severe COVID-19 (Saengsiwaritt et al., 2022). This result may be explained by positive correlation between TMPRSS2 activity and viral load (Okugawa et al., 2023). A genome-wide association study (GWAS) identified that a variant on chromosome 5 at 5q35 (rs60200309-A), close to the dedicator of cytokinesis 2 gene (DOCK2), is associated with severe COVID-19 in patients less than 65 years of age (Namkoong et al., 2022). In addition, genetic variants, such as KLRC2 deletion and reduced HLA-E*0101 levels, have shown to be risk factors for severe COVID-19 (Vietzen et al., 2021). Given the emerging role of genetic factors in the discrepancies in individual susceptibility and severity, genetic predisposition has been considered to a key modulator for SARS-CoV-2 pathogenesis.

In addition to genetic predisposition, the expression of host factors is dynamically and strictly regulated by numerous factors, which might in turn affect disease progression, adding an additional complexity to SARS-CoV-2 pathogenesis. For example, BRD2, an important regulator for ACE2 expression in human lung epithelial cells and cardiomyocytes, has demonstrated to be crucial for the host response to SARS-CoV-2. Correspondingly, BRD2 inhibitors effectively reduced endogenous ACE2 expression and prevented SARS-CoV-2 infection in these cells (Samelson et al., 2022). High mobility group box 1 protein (HMGB1), an evolutionarily conserved chromatin-binding protein that regulates ACE2 expression, is essential for the viral entry of both SARS-CoV-2 and SARS-CoV (Wei et al., 2021). Interestingly, a negative correlation between ACE2 expression and COVID-19 severity has been proposed (Mariappan and Balakrishna, 2020; Ortatatli et al., 2023). The large GTEx data revealed that ACE2 expression is high in Asian females and young people, those who are known to be less susceptible, and even less inflicted by severe or fatal outcome, while it is low in males, further decrease with age and type 2 diabetes mellitus (T2DM), those who are most susceptible to bad outcome (Chen et al., 2020a). The decreased ACE2 expression in males, older individuals, and T2DM patients might contribute to their poor prognosis and high lethality. This phenomenon is likely attributed to the conversion of angiotensin II (Ang-II) to Ang 1-7 by ACE2, which reduces the detrimental effects of Ang-II (Donoghue et al., 2000).

Although GWAS has identified a variety of common genetic variants associated with COVID-19, the functions and mechanisms of these variants are still in the nascent phase. By revealing the genes and pathways involved in SARS-CoV-2 pathogenesis and host-virus interactions, human genome research would not only uncover novel targets for prevention and therapy, but also lay the groundwork for more personalized disease management.

## 5 Host factors in SARS-CoV-2-triggered responses

**Figure 3:**
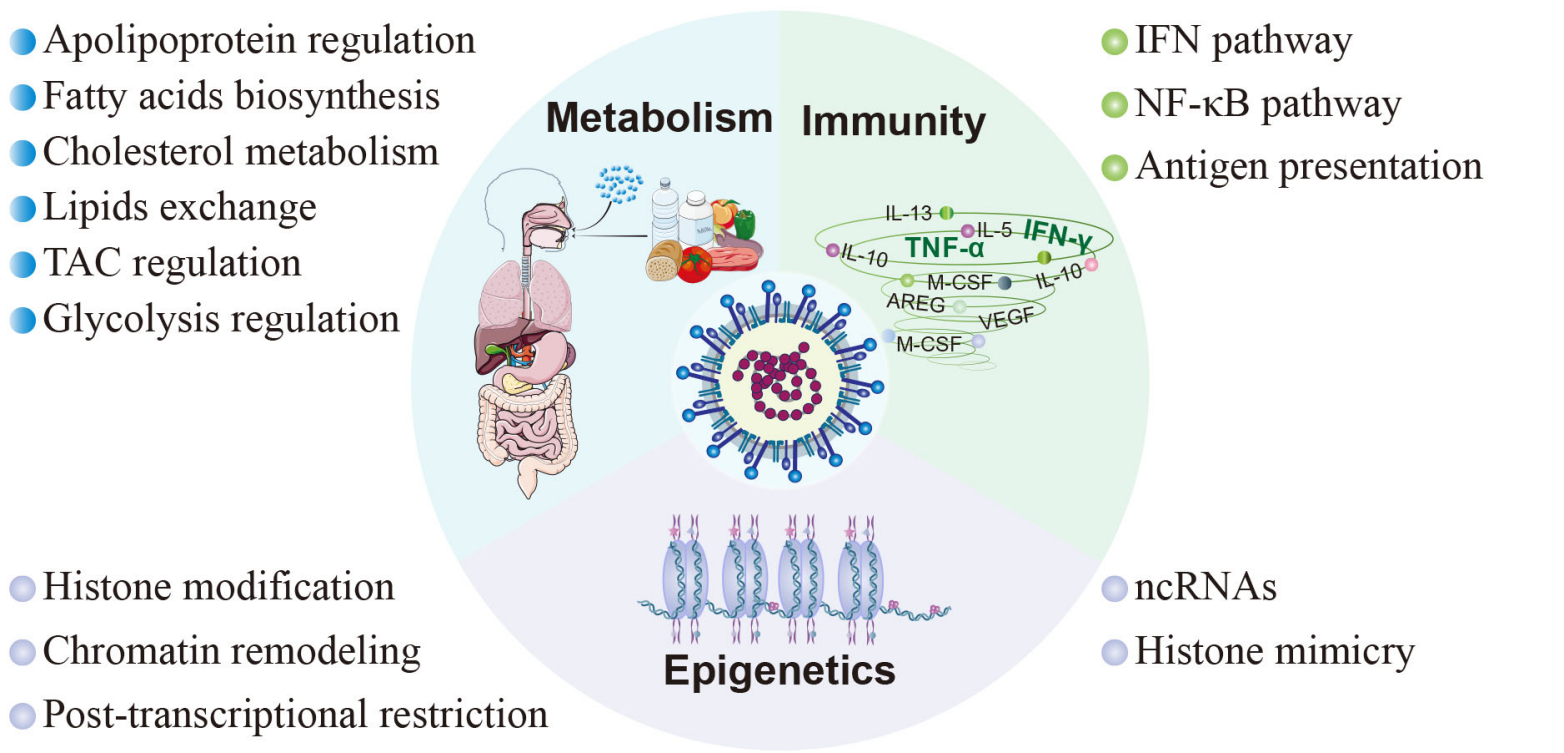
Host factors in SARS-CoV-2 pathogenesis. The pathogenesis subsequent to viral infection involves diverse facets of the host inherent physiological processes, encompassing immunity, metabolism, and epigenetics. In terms of immunity, SARS-CoV-2 modulates the innate immune response through the interferon signaling pathway and NF-κB signaling pathway, and inhibits adaptive immunity by interfering with antigen presentation. As to metabolism, SARS-CoV-2 interferes with cellular metabolism by participating in the regulation of cholesterol metabolism, fatty acid production, tricarboxylic acid cycle, glycolysis, and cell membrane lipid exchange. Moreover, SARS-CoV-2 disrupts host cell epigenetics by means of non-coding RNAs, histone mimicry, chromatin remodeling, and some other mechanisms.

The clinical manifestations of SARS-CoV-2 infection vary from asymptomatic or mild/moderate upper respiratory tract symptoms to life-threatening COVID-19 pneumonia with multi-organ complications (Melenotte et al., 2020). These diverse clinical consequences are closely associated with the host response to SARS-CoV-2, encompassing immune responses, metabolic alterations, and epigenetic modifications (**Figure 3**).

### 5.1 Immune responses

**Figure 4:**
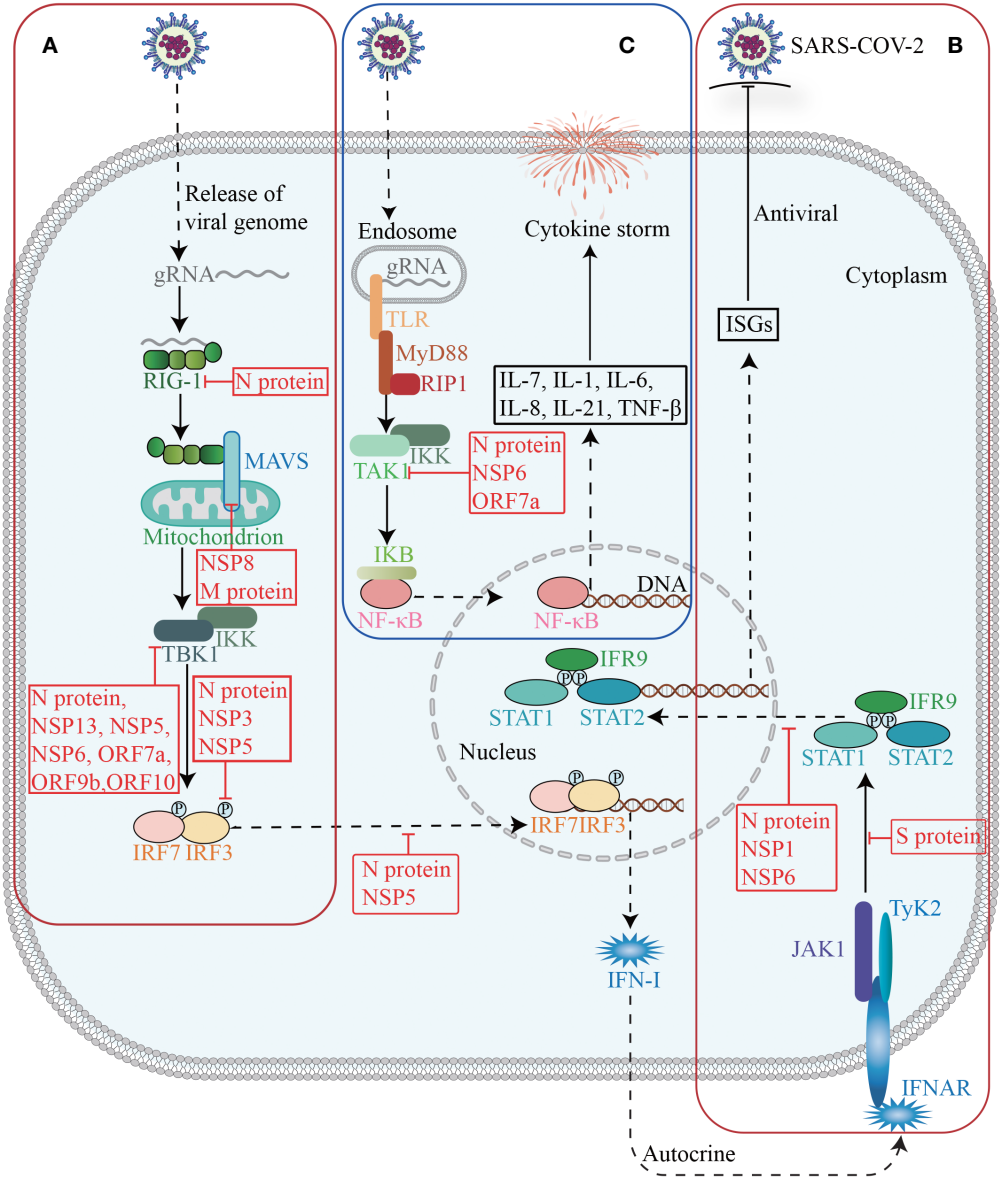
Host factors in immune response. The invasion of SARS-CoV-2 triggers the activation of innate immunity, which subsequently induces the synthesis and secretion of IFN-I **(A)**. Upon binding to its receptor, IFN-I activates the JAK-STAT signaling pathway and subsequently induces the expression of ISGs **(B)**. Activation of TAK1 and IKK triggers the initiation of the NF-κB pathway, leading to a cascade of downstream cytokines and chemokines **(C)**.

The innate immune system serves as the primary defense against SARS-CoV-2, and plays a crucial role in monitoring viral infections, eliminating virus-infected cells, initiating inflammation, and enhancing adaptive immunity. Conversely, SARS-CoV-2 could promote immune evasion through interactions with host proteins, thereby limiting host control and helping itself to replicate and transmit (Diamond and Kanneganti, 2022) (**Figure 4**).

The interferon (IFN) signaling pathway is an essential innate immune defense mechanism against SARS-CoV-2. Upon SARS-CoV-2 infection, signaling cascades are triggered through the recognition of pathogen-associated molecular patterns (PAMPs) by pattern-recognition receptors (PRRs), leading to the production of type I IFN and type III IFN (IFN-I and IFN-III) and the activation of IFN signaling pathways in an autocrine and paracrine manner (Akira et al., 2006; Minkoff and tenOever, 2023), ultimately inducing hundreds of interferon-stimulated genes (ISGs) with various antiviral functions to foster an antiviral state (Schultze and Aschenbrenner, 2021).

Many studies have shown that SARS-CoV-2 employs a multifaceted strategy to suppress the production of IFN-I and IFN-III (Minkoff and tenOever, 2023). Patients with severe and critical COVID-19 have a highly impaired IFN-I response (characterized by the absence of IFN-β and low IFN-α production and activity), which is associated with persistent blood viral load and increased inflammation (Hadjadj et al., 2020). From a holistic view, SARS-CoV-2 could suppress immune responses, by modulating host gene expression, with its NSPs and accessory proteins as essential executors. As mentioned above, NSP1 can widely repress host gene expression through multiple ways. Recent evidence suggests that NSP16 is capable of suppressing host mRNA splicing, whereas NSP8 and NSP9 could interfere protein trafficking to the cell membrane (Banerjee et al., 2020). By interacting with the nuclear pore complex component NUP98-RAE1, ORF6 can either inhibit mRNA nuclear export and thus reshape the host cell proteome, or interfere with nuclear import, specifically the translocation of IRFs and STATs, thereby disrupting IFNs antagonism (Kehrer et al., 2023). These mechanisms independently act and coordinately decrease the expression of host genes, especially IFN-I and IFN-III.

In addition, SARS-CoV-2 NSPs and accessory proteins may interact with essential proteins involved in the production and signaling of IFNs, thereby counteracting their antiviral effects. NSP1 significantly inhibits the phosphorylation and translocation of signal transducer and activator of transcription 1 (STAT1), and thus suppresses the IFN-I signaling (Xia et al., 2020). NSP3 exhibits multifunctionality by cleaving the polypeptide chain translated from the SARS-CoV-2 genome, as well as mediating the removal of polyubiquitin chains and IFN-stimulated gene 15 (ISG15, a ubiquitin-like modifier) from host proteins (Klemm et al., 2020). The latter allows the virus to evade the defenses of the innate immune system. For example, NSP3 can antagonize the conjugation of ISG15 to impair the activation of melanoma differentiation gene 5 (MDA5) (Liu et al., 2021). The deubiquitylation of STING by NSP3 may disrupt the STING-inhibitor of NF-κB kinase-ϵ (IKKϵ)-interferon regulatory factor 3 (IRF3) complex, which is responsible for inducing the production of IFN-β as well as IFN-stimulated cytokines and chemokines (Cao et al., 2023). NSP5 is able to inhibit IFN-I and IFN-III expression by inhibiting the phosphorylation of TRAF family member-associated NF-κB activator binding kinase 1 (TBK1) and IRF3, as well as restraining IRF3 translocation (Zheng et al., 2022). NSP6, NSP13, and ORF9b may directly bind to TBK1 and thus impair phosphorylation-mediated TBK1 activation, while ORF7a can decrease TBK1 expression (Minkoff and tenOever, 2023). Besides, the interaction between ORF10 and STING attenuates the association between STING and TBK1, thereby inhibiting cGAS-STING-induced IRF3 phosphorylation and translocation (Han et al., 2022). Furthermore, NSP8 has shown to disrupt the MDA5-mitochondrial antiviral signaling protein (MAVS) signalosome, resulting in a significant reduction of K63-linked polyubiquitination of MDA5 (Rashid et al., 2022).

SARS-CoV-2 structural proteins are also involved in suppressing the host IFN response. The N protein can competitively bind to TRIM25, an E3 ubiquitin ligase that facilitates RIG-I ubiquitination, and thus attenuate retinoic acid inducible gene I (RIG-I) activation and IFN-I and IFN-III production (Gori Savellini et al., 2021; Minkoff and tenOever, 2023). Besides, the N protein may target RIG-I cofactors, such as G3BP1 and PACT, and quarantine their binding to RIG-I. Furthermore, the N protein is able to inhibit IFN-I and IFN-III expression by suppressing TBK1 and IRF3 phosphorylation and restraining the nuclear translocation of IRF3 (Zheng et al., 2022). Similarly, the M protein has been reported to disrupt the ability of MAVS to establish the necessary scaffolding required for downstream transcription factor activation and to inhibit the function of additional host factors involved in MAVS signaling such as TBK1 (Minkoff and tenOever, 2023). In suppressing IFN signal, the fragments generated through caspase-6 cleavage of the N protein possess the ability to inhibit the production of IFN-β and decrease the expression of representative ISGs, such as interferon induced protein with tetratricopeptide repeats 3 (IFIT3) and 2’-5’-oligoadenylate synthetase 1 (OAS1) (Chu et al., 2022). Both N proteins and NSP6 suppress the phosphorylation of STAT1 and STAT2 as well as the nuclear translocation of STAT1 (Mu et al., 2020; Bills et al., 2023). The S protein can interact with STAT1 to impede its association with JAK1 (Zhang et al., 2021b).

The protective role of IFNs responses in acute SARS-CoV-2 infection is evident, whereas a limited and delayed IFNs response results in heightened production of proinflammatory cytokines and lung parenchymal injury due to infiltration of inflammatory cells. However, there is interpatient variability in the IFN response among individuals with COVID-19, with severe patients exhibiting prolonged production of IFN-I in certain cases and absence of IFN-I expression in others (Lowery et al., 2021). Therefore, while IFN therapy may not confer benefits to all patients, it holds potential utility for specific patient subsets, including those who receive early treatment during infection and individuals exhibiting markedly low IFN responses (Feld et al., 2021; Monk et al., 2021). Further investigations are warranted to reconcile these contradictory findings and establish a universally applicable approach for suitable populations amenable to IFN therapy.

The pathogenesis of COVID-19, particularly in severe cases, is characterized by an excessive systemic inflammatory response known as a cytokine storm, which is closely associated with the activation of the NF-κB signaling pathway (Hadjadj et al., 2020; Kim et al., 2023). Upon activation of PRRs, the adaptor protein myeloid differentiation primary response gene 88 (MyD88) is engaged to initiate the NF-κB pathway, ultimately resulting in the expression of a multitude of cytokines and chemokines (Akira et al., 2006).

SARS-CoV-2 can interact with crucial proteins within the NF-κB pathway, and exacerbate systemic inflammatory response. During viral assemble, after binding to viral RNA, the N protein undergoes robust liquid-liquid phase separation (LLPS), which recruits and enhances the association between the TAK1 and IKK complex, thereby promoting virus-triggered activation of NF-κB signaling (Wu et al., 2021b). NSP6 and ORF7a interact with TAK1 to facilitate NF-κB activation through K63-linked polyubiquitination by TRIM13 and RNF121 (Nishitsuji et al., 2022). NSP14 interacts with inosine-5’-monophosphate dehydrogenase 2 (IMPDH2) and promotes the nuclear translocation of NF-κB p65 (Li et al., 2022a). It is noteworthy that ORF8 can interact with IL-17 receptor A (IL17RA) to regulate the systemic IL-17 signaling pathway, which plays a pivotal role in recruiting monocytes and neutrophils and triggering a cascade of downstream cytokines and chemokines, including IL-1, IL-6, IL-8, IL-21, and tumor necrosis factor beta (TNF-β) (Liu et al., 2017). However, a recent study has provided additional evidence that ORF8 transmits inflammatory signaling in monocytes and macrophages through MyD88 independently of the IL-17R (Ponde et al., 2023).

In addition to the innate immune response, the antiviral defense also involves adaptive immunity, which encompasses B cells and T cells. CD8+ T cells acting as cytotoxic effector cells to eliminate virus-infected host cells. CD4+ T cells can be activated and differentiated into various subsets, exerting commanding and regulatory roles in modulating the activity of other immune cells such as B cells, CD8+ T cells and macrophages, while orchestrating a comprehensive range of immune responses through their production of cytokines and chemokines (Zhu and Paul, 2008). Adaptive immunity can also be compromised by SARS-CoV-2. Recent evidence has revealed a notable absence of lymph node and splenic germinal centers, as well as Bcl-6+ B cells in COVID-19. Additionally, there are also impaired Bcl-6+ T follicular helper cell generation and differentiation, imbalanced T-helper cell polarization, and heterogeneous CD8+ T cells maturation and polarization (Kaneko et al., 2020; Gil-Etayo et al., 2021; Kudryavtsev et al., 2022). Mechanistically, a recent study suggests that viral protein ORF8 could impair the activation of T cells. ORF8 could sequester MHC-I during its transit through the ER, which may hinder viral antigen presentation on the cell surface and reduce recognition and elimination of virus-infected cells by facilitating MHC-I degradation via autophagosome and autolysosome pathways (Zhang et al., 2021d).

These findings clearly demonstrate that SARS-CoV-2 not only hampers the IFNs signaling pathway and antigen presentation to achieve immune evasion, but also triggers the NF-κB pathway, resulting in a severe inflammatory state. Some studies have suggested that the progression and severity of the disease during SARS-CoV-2 infection may be account for these two opposite immune abnormalities. That is, in the early stage of COVID-19, a variety of viral proteins inhibit immune signaling pathways, while in the late stage, the immune response is activated to a certain extent by specific viral proteins, leading to the occurrence of cytokine storm syndrome in patients with severe COVID-19 (Tian et al., 2020). The diverse effects of viral proteins during different periods may be attributed to variations in their proportions (Blanco-Melo et al., 2020), necessitating further investigation into the specific underlying mechanisms.

### 5.2 Epigenetics

Recent findings have suggested that SARS-CoV-2 significantly disrupts epigenetic regulation of host cells through ncRNA, histone mimicry and modification, chromatin remodeling and post-transcriptional restriction (**Figure 3**) (Kee et al., 2022). Among them, ncRNAs, particularly lncRNAs and microRNAs (miRNAs), are the most extensively studied, and can exert regulatory control over immune mechanisms, cellular damage, and virus-related physiological processes by modulating host proteins or viral genomes (Zhang et al., 2021c) (**Table 1**).

**Table 1:** miRNAs in SARS-CoV-2 pathogenesis. .

Name	Origination	Target	Function	References
miR-200c miR-125a-5p miR-1246	Host	ACE2	Inhibit ACE2 expression	(Khan et al; Lu et al., 2020)
miR-98-5P	Host	TMPRSS2	Inhibit TMPRSS2 expression	(Matarese et al., 2020)
miR-155-5p miR-200a-3p miR-26a-5p miR-217 miR-128-3p	Host	SMAD2, SMAD3, SMAD4, SMAD7, TGFBR1	Regulate TGF-β pathway	(Yousefi et al., 2020; Maranini et al., 2022)
miR-29b-3p	Host	IL-4, IL-8	Regulate inflammatory factors	(Centa et al., 2021)
miR-506-3p	Host	NR3C1, SP3, SMARCC1	Inhibit viral replication	(Chow and Salmena, 2020)
miR-325 miR-447b miR-3672	Host	Viral structural proteins	Regulate viral replication	(Sacar Demirci and Adan, 2020)
miR-138-5p miR-196a-5p miR-323a-5p miR-506-3p miR-1202 miR-4758-5p miR-5047 miR-6838-5p	Host	SARS-CoV-2 ORF1a/b	Inhibit the cleavage and translation of ORF1a/b	(Chow and Salmena, 2020)
miR-146	Host	CLEC5A, STAT1	Regulate inflammatory factors and responses to IFNs	(Jafarinejad-Farsangi et al., 2020; Roganović, 2021)
miR-9 miR-34a-5p miR-98 miR-214 miR-223	Host	NF-kB, JAK-STAT	Regulate inflammatory factors	(Amini-Farsani et al., 2021; Donyavi et al., 2021)
MR147-3p	Virus	TMPRSS2 enhancer	Promote TMPRSS2 expression	(Zhang et al., 2021c)
MD2-5p MR147-3p	Virus	CHAC1, RAD9A	Reduce apoptosis	(Zhang et al., 2021c)
MR66-3p MR147-5p MR198-3p MR328-5p MR359-5p MR385-3p	Virus	the enhancer of CXCL16, ARRB2, TNF-α, ADAR, MYH9, RARA, and TGFBR3	Regulate immune system and inflammatory response	(Zhang et al., 2021c)

The infection of SARS-CoV-2 triggers an upregulation of host miRNAs, which subsequently targeting numerous host genes. The S protein stimulates the generation and release of exosomes containing miR-148a and miR-590, which are internalized by microglia to inhibit the expression of ubiquitin specific peptidase 33 (USP33) and downstream IRF9 in the nervous system. These miRNAs play a role in the regulation of TNFα, NF-κB, and IFN-β pathways, resulting in excessive activation of microglia and subsequent damage to the central nervous system (Mishra and Banerjea, 2021). In cardiomyocytes, miR-200c plays an antiviral role in regulating the entry of SARS-CoV-2 by targeting the 3’-UTR of ACE2 mRNA and subsequently reducing its expression (Lu et al., 2020). In endothelial cells, miR-98-5p directly targets and represses TMPRSS2 (Matarese et al., 2020). RNA-seq and bioinformatics analysis reveal extensive complementarity between multiple human miRNAs and the functional RNAs of SARS-CoV-2 (Sacar Demirci and Adan, 2020; Hill and Lukiw, 2022), such as miR-447b and the RNA of the S protein, miR-3672 and the RNA of the E protein, and miR-325 and the RNA of the M protein. However, the epigenetic regulation of viral proteins by human miRNAs still needs further investigation and verification. The expression profiles of human miRNAs exhibit significant tissue-specific and inter-individual variations (Hill and Lukiw, 2022). The tissue-specific expression patterns of miRNAs may partially contribute to the variations in susceptibility and pathological response to SARS-CoV-2 across different tissues, while the significant differences in miRNA abundance and functionality among individuals may result in disparities in immune response and disease severity.

On the other hand, SARS-CoV-2 also encode its own miRNAs. miRNAs derived from SARS-CoV-2 have the potential to modulate the host immune system and inflammatory response (Zhang et al., 2021c). For instance, MR385-3p may could bind to 5’-UTR of TGFBR3, a key receptor of immune system, and MR66-3p may could bind to the enhancer of TNF-α, an important cytokine in the cytokine storm. While a viral miRNA-like small RNA (CoV2-miR-O7a) has been identified to target basic leucine zipper ATF-like transcription factor 2 (BATF2), thereby modulating the IFN signaling (Pawlica et al., 2021). In addition, MD25p and MR147-3p target the apoptosis-related gene CHAC1 and RAD9A respectively, involved in the host cellular apoptotic response to viral infection (Zhang et al., 2021c). Given the important role of host and viral encoded miRNAs in COVID-19 pathogenesis, the exploration for specific miRNAs with potential therapeutic benefits against SARS-CoV-2 can be regarded as a promising strategy to mitigate the severity of COVID-19. However, since a single miRNA can regulate a large number of mRNAs, high doses of a single miRNA *in vivo* might lead to severe off-target adverse effects. Therefore, it may be an effective therapeutic strategy to clarify the target of various miRNA and use multiple low-dose miRNAs in combination.

LncRNAs can modulate gene regulation at multiple levels, including chromatin remodeling, transcriptional regulation, and post-transcriptional processing Ernst and Morton, 2013). An increasing number of lncRNAs have been implicated in SARS-CoV-2 infection and subsequent host responses (Qiu et al., 2018). For example, the expression of IL-6 can be regulated by several lncRNAs, including TSLNC8, MALAT1, NEAT, CAIF and HOTAIR, through various pathways such as JAK/STAT, NF-κB, hypoxia inducible factor-1α (HIF-1α), and mitogen-activated protein kinase (MAPK) (Serpeloni et al., 2021). More recently, it has shown that the SARS-CoV-2 N protein hinders the recruitment of DNA damage response factor 53BP1 at DNA double-strand breaks by competing with the binding of damage-induced lncRNAs, and thus impedes DNA repair, adding another complexity to lncRNA regulation on SARS-CoV-2 pathogenesis (Gioia et al., 2023). Notably, a multitude of lncRNAs are differentially expressed in response to viral infection. A deeper understanding of the change in lncRNA transcriptome in infected cells, as well as their effects on interactions between host and virus, could eventually facilitate the identification of novel therapeutic targets and the development of new diagnostic biomarkers and therapeutic treatments.

In addition to ncRNAs, SARS-CoV-2 can impact other forms of epigenetic modifications. ORF8 functions as a histone mimic of the ARKS motifs in histone H3 to disrupt host cell epigenetic regulation (Kee et al., 2022). NSP5 can interact with histone deacetylase 2 (HDAC2) to modulate HDAC2 nuclear entry and subsequent inflammation and IFN response (Xu et al., 2019; Gordon et al., 2020). The E protein may bind to bromodomain containing 2 (BRD2) and BRD4 (Gordon et al., 2020). and thus influence their interaction with acetylated histones and gene transcription (Faivre et al., 2020). Interestingly, a multitude of transcription factors, such as JUN, and zinc finger and BTB domain containing 20 (ZBTB20), are responsible to SARS-CoV-2 infection (Alexander et al., 2021). It is evident that post-transcriptional restriction of JUN can impede the induction of IFNs, while ZBTB20 reduction may delay the immune response. These findings suggest that SARS-CoV-2 hampers the host’s immune response through diverse epigenetic regulatory mechanisms.

### 5.3 Metabolism

The prediction of protein targets for SARS-CoV-2 reveals a significant enrichment in metabolic processes related to lipids, amino acids, and glucose (Dey et al., 2020), suggesting a profound impact on host metabolism.

In addition to be the main receptor for SARS-CoV-2, ACE2 can regulate the expression of many critical genes involved in glucose and lipid metabolism, such as glucose-6-phosphatase, catalytic (G6PC), glucose transporter 2 (GLUT2), peroxisome-proliferator-activated receptor coactivator (PGC)-1α, peroxisome proliferators-activated receptors α (PPARα), and PPARγ, suggesting a role in SARS-CoV-2-induced metabolic dysregulation (Li et al., 2022b). Recently, SARS-CoV-2 infection has been shown to increase the expression of RE1-silencing transcription factor (REST), which regulates hundreds of genes including many metabolic factors, eventually leading to abnormal metabolism of glucose and lipids (He et al., 2021).

Genome-wide CRISPR knockout screening studies suggest cholesterol metabolism as the most significant pathways associated with SARS-CoV-2 infection (Schneider et al., 2021). Several lines of evidence indicate that cholesterol may facilitate viral infection. Putative cholesterol recognition amino acid consensus motifs have been recently identified in the S protein, and antibodies that block these cholesterol-binding sites significantly hindered SARS-CoV-2 entry (Wei et al., 2020; Baier and Barrantes, 2023). Amlodipine, the drug that disrupts the cholesterol biosynthesis pathway, is sufficient to reduce SARS-CoV-2 infection (Daniloski et al., 2021). Similarly, 25-hydroxycholesterol that activates acyl-coA: cholesterol acyltransferase (ACAT) and leads to cholesterol depletion on the plasma membrane can prevent SARS-CoV-2 entry (Wang et al., 2020c). Niemann-Picker intracellular cholesterol transporter 1 (NPC1), a regulator of endosomal/lysosomal vesicle transport and cholesterol efflux, is important for the cytoplasmic entry of the viral genome, and its mutations or deletions exhibit an anti-viral effect (Vial et al., 2021). The high-density lipoprotein (HDL) components could bind to S1 subunit of the S protein, and thus enhance the HDL scavenger receptor B type 1 (SR-B1)-mediated virus attachment on cell surface and subsequent virus uptake (Wei et al., 2020). Transmembrane protein 41B (TMEM41B) may contribute to the formation of viral replication complex via mobilization of cholesterol and other lipids to facilitate host membrane expansion and curvature (Ji et al., 2022). Interestingly, apolipoprotein E (APOE), a key factor in cholesterol metabolism, could interact with a key docking site for SARS-CoV-2 S protein on ACE2, thereby reducing ACE2/S-mediated viral entry into cells. Taken together, as a conserved cellular mechanism exploited by viruses for host cell entry and replication, the lipid metabolism pathway represents a crucial research avenue in the development of host-targeted antiviral drugs.

From another aspect, SARS-CoV-2 infection disrupts normal lipid metabolism. General dyslipidemia was observed by targeted or untargeted lipidomic analyses of plasma, particularly in severe COVID-19 patients (Casari et al., 2021). These abnormally metabolized lipids not only have a structural role, but also regulate numerous signaling pathways and immune responses (Hannun and Obeid, 2018). Mechanistically, SARS-CoV-2 could interact with sterol regulatory element binding transcription factor chaperone (SCAP), and membrane-bound transcription factor peptidase, site 1 and 2 (MBTPS1 and MBTPS2), as well as regulators of the cholesterol biosynthesis pathway, to modulate the biosynthesis of fatty acids and cholesterol (Wang et al., 2021b). The viral protein NSP6 can upregulate many cholesterols metabolism-related proteins, such as 7-dehydrocholesterol reductase, cytochrome p450 family 51 subfamily A member 1, isopentenyl-diphosphate delta 1somerase 1, and squalene epoxidase (Stukalov et al., 2021). Besides, the S protein has the ability to extract lipid components from HDL, leading to a modification in the HDL functionality for lipid exchange with model cellular membranes (Correa et al., 2021). As strongly altered in COVID-19 and correlate with the severity of the disease, specific lipid components may serve as potential biomarkers for the identification and severity classification of COVID-19.

Glucose metabolism is also disrupted by SARS-CoV-2 infection. COVID-19 can result in new-onset hyperglycemia or worsening glycemic control (Conte et al., 2024), and fasting plasma glucose levels can even predict the mortality of COVID-19 patients (Cai et al., 2020). Consistently, SARS-CoV-2 infection is also influenced by the host glucose metabolism. Blood glucose levels appear to be positively associated with viral load (Codo et al., 2020; Zhu et al., 2020; Garreta et al., 2022). Elevated blood glucose levels can increase the stability of ACE2 mRNA, leading to up-regulation of ACE2 expression and promoting the entry of SARS-CoV-2 into cells (Garreta et al., 2022; Vargas-Rodriguez et al., 2022). Like lipid metabolism, host glucose metabolism is regulated by SARS-CoV-2. For example, the interaction between NSP14 and sirtuin 5 facilitates the succinylation of host proteins including several key enzymes of the tricarboxylic acid cycle (TAC), thereby inhibiting cellular metabolic pathways (Liu et al., 2022c). SARS-CoV-2 also modifies proteins related to glycolysis. Although the underlying mechanisms remain unclear, SARS-CoV-2 infection leads to an excessive activation of glycolysis, resulting in a significant consumption of glucose, pyruvate, glutamine, and alpha-ketoglutaric acid. Notably, the blockade of glutaminolysis has been shown to impair both viral replication and the inflammatory response (de Oliveira et al., 2022).

Altogether, glucose and lipid metabolism are essential for the cell to conduct fundamental physiological functions. The impact of SARS-CoV-2 on glucose and lipid metabolism, and its emerging roles in other crucial metabolic pathways, would contribute to the occurrence and progression of COVID-19, as well as its complications and sequelae, particularly metabolic diseases.

## 6 Long-term effects of COVID-19

The implementation of public health policies, vaccination, and comprehensive acute therapies have led to a decrease in COVID-19 mortality. However, the aftermath of this pandemic has given rise to post-acute sequelae, commonly known as long COVID or post-COVID conditions (PCC) (Brightling and Evans, 2022; Stafie et al., 2022). It has estimated that long COVID, a frequently debilitating illness, affects at least 10% of individuals surviving from COVID-19 (Davis et al., 2023). The majority of these individuals experience persistent and chronic symptoms weeks to years after the initial infection, such as fatigue, dyspnea, joint pain, chest pain, and other conditions (Lai et al., 2023). In addition, COVID-19 may often lead to more enduring and wider-ranging long-term adverse effects, including cardiovascular, cerebrovascular, metabolic, and nervous system diseases (Xie and Al-Aly, 2022; Xie et al., 2022). A recent study estimated the risks associated with 80 prespecified post-acute sequelae of COVID-19 over a period of 2 years, and found that certain post-acute sequelae, particularly those related to gastrointestinal, musculoskeletal, and neurologic systems, exhibit a prolonged risk horizon extending beyond 2 years or even longer (Bowe et al., 2023). Therefore, the knowledge on long COVID-19 may provide evidence for preventing and treating the sequelae, and offering valuable insights into its interconnectedness with other diseases.

**Figure 5:**
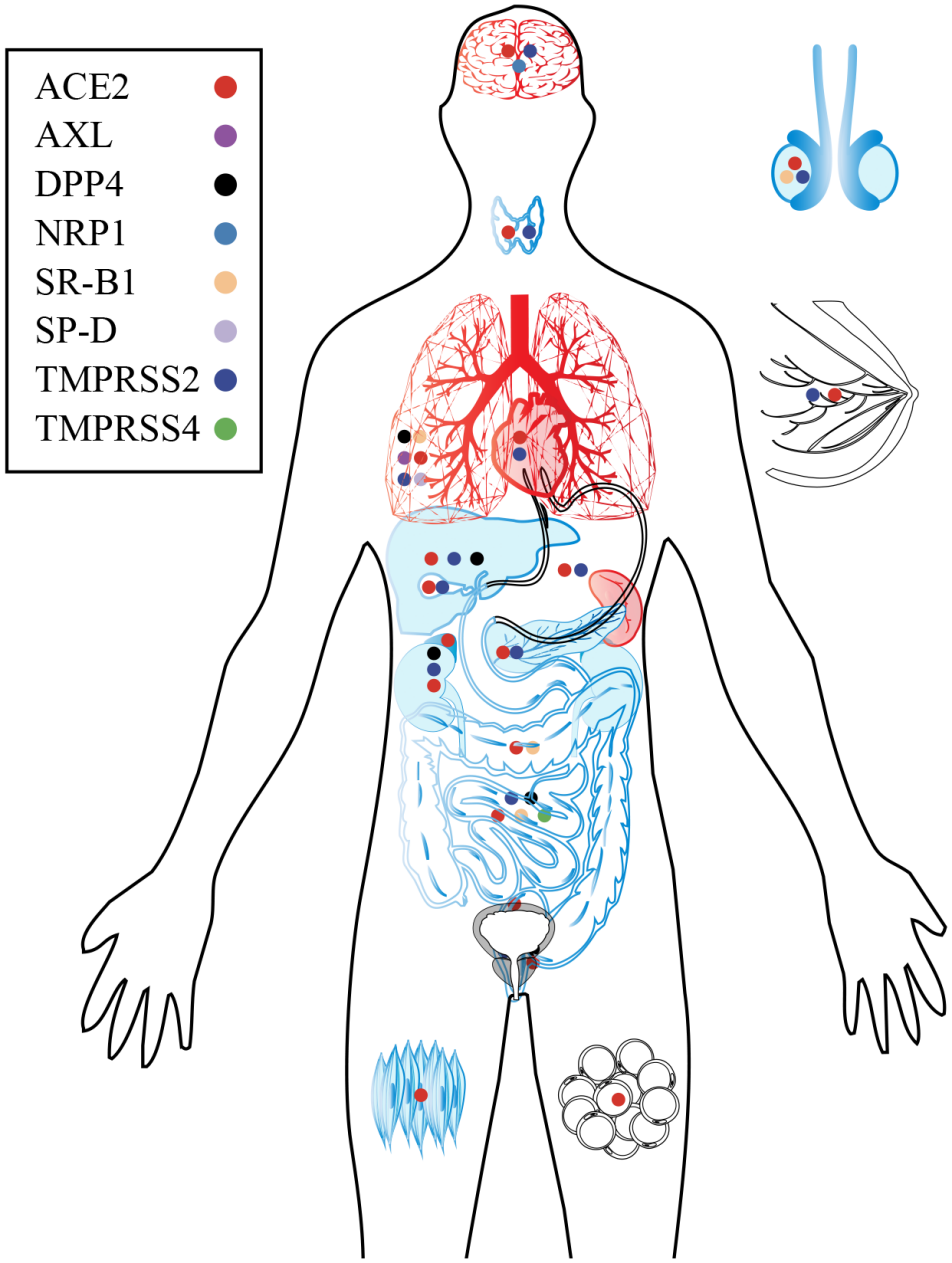
Target organs and major receptors of SARS-CoV-2. As the primary receptor for SARS-CoV-2, ACE2 is widely distributed throughout human organs and tissues. Additionally, specific receptors in certain organs and tissues can also facilitate viral infection and subsequent damage. The organs are depicted in black, blue, and red to indicate different aspects. Black represents the distribution of SARS-CoV-2 receptors in the organ; Blue of viral infection and replication within the organ; Red indicates damage caused by the virus.

**Figure 6:**
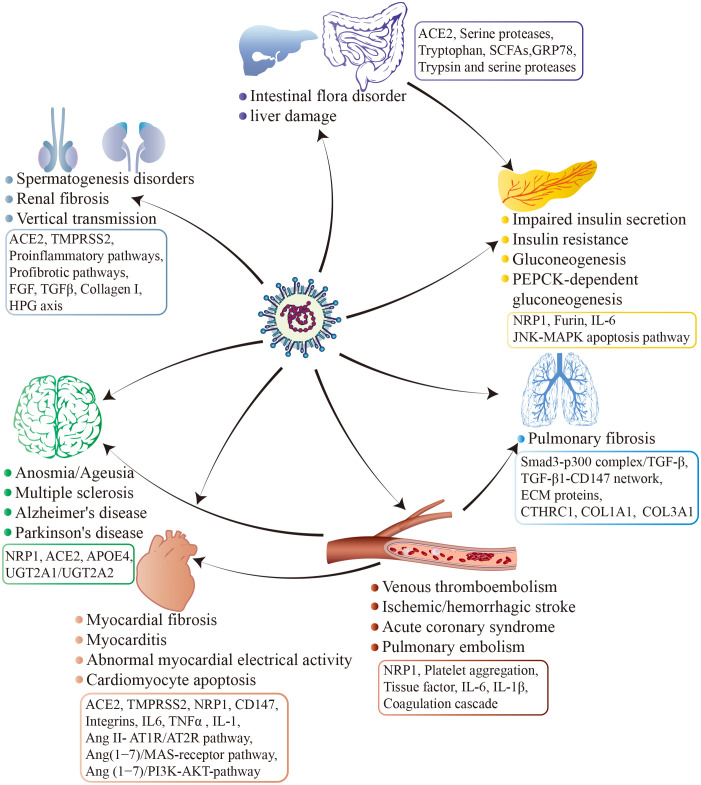
Long-term effects in COVID-19 survivors. By interacting with host factors, SARS-CoV-2 manipulates the intricate network of molecular mechanisms and disrupts various normal physiological mechanisms and processes in the host. Due to tissue specificity, SARS-CoV-2 potentially invades various organs via distinct cell surface receptors, trigger diverse signaling pathways, induce corresponding pathological damage, and ultimately result in disparate complications and sequelae across different organ systems. In addition to surface-level manifestations, SARS-CoV-2 infection may pose a significant risk factor for the development of long-term chronic conditions such as cardiovascular and cerebrovascular diseases, metabolic disorders, and neurodegenerative ailments.

The infection of SARS-CoV-2 is contingent upon the presence of specific receptors and proteases on cell surface (Dong et al., 2020). These receptors and proteases are found in various cell types throughout the human body, including the oral and nasal mucosa, lung, heart, gastrointestinal tract, liver, kidney, spleen, brain, as well as arterial and venous endothelial cells (Pennisi et al., 2020; Sungnak et al., 2020; Nakamura et al., 2021; Qi et al., 2021; Martínez-Mármol et al., 2023), highlighting that SARS-CoV-2 may affect almost every organ, leading to acute organ damage and long-term sequelae (**Figure 5**). Due to the unique characteristics of various tissues and organs, the symptoms and potential long-term effects of COVID-19 vary considerably. However, major contributors are consistent and proposed to include abnormally altered immune system, circular system dysfunction, multi-organ damage, and neuroinflammation. Although mechanistic studies are generally in their early stages, it is undeniable that host factors play an indispensable role (Davis et al., 2023) (**Figure 6**).

### 6.1 The respiratory system

The respiratory system is the hardest hit by SARS-CoV-2, and the severity of COVID-19 is closely correlated with the extent of lung damage. SARS-CoV-2 causes permanent lung damage through three interacting mechanisms, namely acute respiratory distress syndrome (ARDS) with diffuse alveolar damage (DAD), diffuse thrombotic alveolar microvascular occlusion, and airway inflammation (Calabrese et al., 2020; Xu et al., 2020b). In addition, SARS-CoV-2 may activate some pathological pathways and pave the way for long-term sequelae.

Chronic pulmonary fibrosis is the most common and severe respiratory sequelae of COVID-19, with an estimated one-third of survivors infected with SARS-CoV-2 developing significant fibrotic changes in their lungs (Wang et al., 2020a). SARS-CoV-2 induces pulmonary fibrosis by upregulating pro-fibrotic molecules and extracellular matrix (ECM) proteins (Zhao et al., 2008). The expression of transforming growth factor beta 1 (TGFB1) and connective tissue growth factor (CTGF) was increased in alveolar epithelial cells of COVID-19 patients (Xu et al., 2020). Previous studies suggest that the N protein of SARS-CoV may enhance the transcriptional responses of TGF-β by interacting with Smad3 and promoting the formation of the Smad3-p300 complex (Zhao et al., 2008). Of note, TGF-β can potently promote fibrosis by modulating the ECM proteins, particularly fibronectin (FN), which are abundantly expressed in lung epithelial cells (Liu et al., 2019; Xu et al., 2020a). CD147 is a universal receptor of SARS-CoV-2 (Wang et al., 2020b), as mentioned above. Interestingly, it has recently shown that CD147 may contribute to fibroblast activation induced by SARS-CoV-2 through a TGF-β1-CD147 self-sustaining network (Wu et al., 2022), Furthermore, many COVID-19 patients exhibit an increased frequency of pathological fibroblasts (pFBs) (Melms et al., 2021). These pFBs are crucial for the development of pulmonary fibrosis, as indicated by robust increases in collagen triple helix repeat containing 1 (CTHRC1) that regulates collagen matrix deposition, and the pathogenic ECM components such as collagen type I alpha 1 chain (COL1A1) and COL3A1 (Tsukui et al., 2020). It is evident that SARS-CoV-2 infection activates various pro-fibrotic pathways, making pulmonary fibrosis as one of challenging sequelae that we need to focus on.

### 6.2 The cardiovascular system

Cardiac symptoms occurring after the acute phase of COVID-19 or in recovered patients can be classified into two main categories: cardiovascular symptoms and myocardial symptoms (Zheng et al., 2020; Tajbakhsh et al., 2021). The former is primarily caused by endothelial-microvascular injury, while the latter is mainly a result of myocardial injury. Both direct infection and dysregulation of systemic inflammation can lead to endothelial impairment (Monteil et al., 2020). Disruption of the subendothelial barrier facilitates the release of tissue factors and triggers the production of multiple components in the coagulation cascade, including fibrinogen alpha chain (FGA), fibrinogen beta chain (FGB), and fibrinogen gamma chain (FGG) (Yan et al., 2021), ultimately leading to prothrombin-to-thrombin conversion. In addition, SARS-CoV-2 could interact with platelets, which are rapid responders to the presence of pathogens, thereby promoting thrombosis and intravascular coagulation (Sciaudone et al., 2023). Endothelial dysfunction, platelet aggregation and the activation of the coagulation cascade contribute to the prothrombotic environment and diffuse intravascular coagulation, resulting in conditions such as pulmonary embolism, acute coronary syndrome, ischemic/hemorrhagic stroke, or venous thromboembolism (Colling and Kanthi, 2020; Jose and Manuel, 2020; Wang et al., 2020d). A recent study has demonstrated the involvement of macrophages in SARS-CoV-2-induced arterial endothelial injury. SARS-CoV-2 can infect foam cells through the NRP1 receptor, triggering a strong inflammatory response by the release of interleukins IL-6 and IL-1β. This plaque inflammation in turn may contribute to the development of acute cardiovascular complications and increase the long-term risk for cardiovascular events (Eberhardt et al., 2023).

The potential for long-term cardiac complications has been emphasized, as evidenced by myocardial fibrosis and myocarditis in up to 78% and 60% of recovered COVID-19 patients, respectively (Puntmann et al., 2020; Chung et al., 2021). Mechanisms of myocardial cell injury may encompass direct viral infection, hypoxia-induced apoptosis and cellular damage mediated by cytokine storm (Jose and Manuel, 2020). *In vivo* studies have demonstrated that cardiomyocytes express the requisite components for SARS-CoV-2 infection, including ACE2, TMPRSS2, NRP1, CD147 and integrins (Abdi et al., 2022). *In vitro* studies have also demonstrated that the virus can infect and induce cytotoxic effects in human cardiomyocytes (Bojkova et al., 2020). A post-mortem biopsy also found evidence of SARS-CoV-2 infecting cardiomyocytes (Nakamura et al., 2021). ACE2 in cardiomyocytes exerts anti-inflammatory and cardioprotective functions through various pathways, including the angiotensin II (Ang II)- angiotensin II type 1 receptor (AT1R)/angiotensin II type 2 receptor (AT2R) pathway (Paradis et al., 2000; Lambert et al., 2005; Wakui et al., 2010), the Ang (1−7)/MAS-receptor pathway (Santos et al., 2006), and the Ang (1−7)/phosphatidylinositol 3-kinase (PI3-K)-protein kinase B (AKT)-pathway (Vecchione et al., 2005). SARS-CoV-2 infection can lead to the reduction of ACE2 on the cell surface, thereby impairing its physiological effects and causing negative consequences. Furthermore, abnormalities of these pathways may contribute to the initiation of a cytokine storm, which, in combination with overactive immune responses, would exacerbate damage to infected cardiomyocytes (Gao et al., 2021). For instance, excessive inflammatory factors (IL6, TNFα and IL-1) can disrupt myocardial ion channels, leading to abnormal myocardial electrical activity and even cardiomyocyte apoptosis (Lazzerini et al., 2019), posing a hidden risk for future cardiac failure (Hafiane, 2020). Particularly, although evidence of direct SARS-CoV-2 infection of cardiomyocytes is available, other possibilities that mediate these outcomes, such as PAMPs, cannot be ignored. In conclusion, there have been reports of cardiac-related symptoms and signs in long COVID (Huang et al., 2020; Puntmann et al., 2020; Clark et al., 2021; Rajpal et al., 2021). Further exploration on the potential for SARS-CoV-2 to infect and cause damage to cardiovascular endothelial cells and cardiomyocytes would improve our understanding of the long-term effects of COVID-19 on the heart.

### 6.3 The nervous system

Both the central nervous system (CNS) and peripheral nervous system (PNS) sustain long-term impairment from SARS-CoV-2 (Singal et al., 2020; Ostermann and Schaal, 2023). While the incidence of neurological complications associated with COVID-19 during the acute and subacute phases ranges from 35% to 85%, our understanding of the post-acute neurological sequelae of COVID-19 remains limited (Nolen et al., 2022). SARS-CoV-2 has the ability to impair mitochondria directly and induce apoptosis, or necrosis in neurocytes (Singh et al., 2020). Due to the absence of regenerative capabilities in neuronal cells, necrosis and apoptosis of infected neurons can cause irreversible harms to the CNS (Nainu et al., 2017). In addition to the direct damage, the inflammatory responses triggered by SARS-CoV-2, both systemically and in the brain, may activate long-term mechanisms that heighten the risk of neurological diseases among infected individuals (De Felice et al., 2020).

The most common symptoms in the nervous system are anosmia and ageusia. NRP1, one of the alternative receptors for SARS-CoV-2, is highly expressed in the olfactory epithelium, olfactory tubercles, and paraolfactory gyri, which might play a role in the infection and damage caused by SARS-CoV-2 at these sites and the occurrence of anosmia (Davies et al., 2020; Vial et al., 2021). Besides, a multi-ancestry GWAS has identified a correlation between the UDP glucuronosyltransferase family 2 member A1/A2 (UGT2A1/UGT2A2) and COVID-19-related anosmia and ageusia. Both UGT2A1 and UGT2A2 are enriched in the olfactory epithelium and govern odorant metabolism (Shelton et al., 2022), and it is of interest for future study to delineate the mechanisms underlying their contributions to anosmia and ageusia.

The neuronal damage caused by SARS-CoV-2 may be the driving force for chronic degenerative diseases, such as multiple sclerosis (MS) (Singal et al., 2020). There is evidence suggesting the presence of coronavirus infection in both brain tissue and cerebrospinal fluid among patients with MS, such as MHV A59 and HCV OC43 (Murray et al., 1992). Moreover, the symptoms of MS share similarities with the neurological changes induced by SARS-CoV-2, such as inflammation and demyelination in the brain and spinal cord (Palao et al., 2020). Neuroinflammatory response, synaptic pruning, and neuron loss serve as the pathological foundation of Alzheimer’s disease (AD) and Parkinson’s disease (Wang et al., 2020a). SARS-CoV-2 can expedite these processes by widely infecting the nervous system through ACE2 that is extensively expressed in various regions of the CNS (Wang et al., 2020a; Merello et al., 2021). It is worth mentioning that APOE4, the most potent risk factor for late-onset AD, can interact with ACE2 and enhance astrocytic response to SARS-CoV-2 infection (Wang et al., 2021a; Zhang et al., 2022a). There may be a potential epidemiological correlation between APOE4-mediated AD and COVID-19. In addition, SARS-CoV-2 is capable of transsynaptic transfer and retrograde or anterograde movement along axons (Li et al., 2012; Li et al., 2020b), thereby enabling a gradual and diffuse infection of the entire nervous system. This may result in patients developing degenerative neurological disorders months or even years after the acute phase of infection.

### 6.4 The digestive system

The common gastrointestinal symptoms of long COVID include nausea, vomiting, diminished appetite, heartburn, abdominal discomfort and constipation (Meringer and Mehandru, 2022). Potential mechanisms underlying these symptoms may include persistent viral antigen presence in mucosal tissues triggering ongoing host responses, changes in the microbiome induced by SARS-CoV-2 infection, aftereffects or sequelae of host immune responses to the SARS-CoV-2 virus, persistent activation or alteration of immune cells and inflammatory signaling pathways, or postinfectious irritable bowel syndrome (Summa and Hanauer, 2023).

Patients with COVID-19 exhibited significant changes in their fecal microbiomes, characterized by an increase in opportunistic pathogens and a decrease in beneficial commensals (Zuo et al., 2020; Guo et al., 2021). Interestingly, distinct microbiota patterns were associated with different post-COVID-19 syndromes. Specifically, respiratory symptoms showed a positive correlation with opportunistic pathogens, while neuropsychiatric symptoms were accompanied by changes in nosocomial pathogens (Liu et al., 2022b). Thus, the modification of the gut microbiota caused by SARS-CoV-2 may be one of the major causes of long COVID syndrome. In details, the replication and transmission of SARS-CoV-2 in the intestinal may affect the absorption of tryptophan, which further leads to the reduction of probiotics such as Lactobacillus and Bifidobacterium. Disruption of the structure and normal metabolism of gut microbiota results in heightened inflammatory responses due to the decrease of short chain fatty acids (SCFAs), metabolites produced by gut microbiota that possess a certain inhibitory effect on cytokine storms (Zhang et al., 2016; Lei et al., 2021a).

Emerging evidence suggests a potential association between SARS-CoV-2 and hepatic injury, as liver dysfunction has been observed in 14-53% of COVID-19 patients (Zhang et al., 2020). Hepatic involvement in COVID-19 may be attributed to various factors, such as direct viral-induced cellular destruction, aberrant immune responses, systemic inflammatory response syndrome, or drug-induced liver injury (Jothimani et al., 2020). These factors collectively contribute to the development of hepatic steatosis, cholestasis, bile duct alterations, and hypoxic hepatitis (Nardo et al., 2021). ACE2 is highly expressed in hepatocytes, bile duct cells, and hepatic endothelial cells (Hamming et al., 2004). Additionally, it is increased in hepatocytes during liver fibrosis or cirrhosis (Huang et al., 2009), potentially exacerbating the tropism of SARS-CoV-2 in the injury liver. The chaperone glucose-regulated protein 78 (GRP78) is abundant in hepatocytes and can function as an entry factor for SARS-CoV-2 (Carlos et al., 2021). Moreover, hepatocytes express a multitude of trypsin and other serine proteases, which could potentially activate the S protein and enhance its affinity for receptors (Letko et al., 2020). Furthermore, the infection of SARS-CoV-2 in cholangiocytes results in a reduction in tight junction proteins such as claudin 1 (Zhao et al., 2020), indicating that compromised barrier function might promote the leakage of potentially toxic bile into adjacent liver parenchyma and contribute to liver injury (Nardo et al., 2021). The gut microbiota and liver play pivotal roles in digestion, metabolism, and immunity within the human body. Hence, any alterations to the gut microbiota as well as potential hepatic damage caused by SARS-CoV-2 will have profound and enduring implications for overall human health.

### 6.5 The urogenital system

Kidney diseases are also a matter of concern as long-term sequelae of COVID-19. A large cohort study revealed that recovered COVID-19 individuals exhibited an increased risk of acute kidney injury, decline in eGFR, kidney failure and major adverse kidney events, particularly the rate of kidney failure was nearly three times higher compared to those without a known infection (Bowe et al., 2021). Renal fibrosis may be the main cause of renal sequelae caused by SARS-CoV-2 (Jansen et al., 2022). ACE2 and TMPRSS2 are widely expressed in the kidney, which enables direct infection of various kidney cell types by SARS-CoV-2, including tubular epithelium, podocytes, and parietal epithelial cells (Müller et al., 2021; Omer et al., 2021). SARS-CoV-2 infection of renal epithelial cells led to the upregulation of proinflammatory and profibrotic signaling pathways, accompanied by deposition of extracellular mechanisms (Jansen et al., 2022). The signaling of fibroblast growth factor (FGF), TGFβ, and collagen I from proximal tubular cells and podocytes to mesenchyme cluster 1 (PDGFRa/b) implies the potential association with fibrosis development (Kuppe et al., 2021; Jansen et al., 2022).

The underlying impact of SARS-CoV-2 on the reproductive system could result in compromised gametes and have repercussions for future generations (Ata et al., 2023). COVID-19 in males predominantly leads to persistent compromised reproductive functions, even after recovery (Dai et al., 2023). The high expression level of ACE2 in various testicular cells, including vas deferens cells, spermatogonial cells, stromal cells, and sertoli cells, renders the testes highly susceptible to SARS-CoV-2 infection (Fu et al., 2020; Hikmet et al., 2020; Wang and Xu, 2020; Qi et al., 2021; Ribeiro et al., 2023). Several *in vitro* and *in vivo* studies demonstrated the susceptibility of testicular cells to SARS-CoV-2 (Campos et al., 2021; Mahé et al., 2023; Chen et al., 2023a; Li et al., 2023b). Due to the physiological barriers, SARS-CoV-2 could potentially persist in the semen of recovered patients (Li et al., 2020a). SARS-CoV-2 has been demonstrated to disrupt spermatogenesis and impede sperm maturation (Shen et al., 2020). Mechanistically, SARS-CoV-2 impairs male fertility by interfering with the hypothalamus–pituitary–gonadal (HPG) axis, downregulating the expression of spermatogenesis-related genes, and increasing oxidative stress (OS) (Dai et al., 2023). Regarding females, studies have provided confirmation of the presence of SARS-CoV-2 in placenta, thereby supporting the theory of vertical transmission of SARS-CoV-2 (Sharma et al., 2021). Although vertical transmission of SARS-CoV-2 requires demanding conditions (Sharma et al., 2021), the hypothesis has been substantiated by a case demonstrating transplacental transmission from a SARS-CoV-2 positive mother to her neonate (Vivanti et al., 2020). The impact of SARS-CoV-2 infection on menstruation has raised concerns regarding its potential effects on women’s fertility (Cherenack et al., 2022). A recent prospective cohort study indicates that there was a short-term (60-day) decline in male fertility following SARS-CoV-2 infection, but female fertility remained unaffected (Wesselink et al., 2022). Nevertheless, a retrospective study reports that patients who were infected by SARS-CoV-2 before the pregnancy delivered at the lowest gestational age, had infants with the lowest birth weight, had the most pregnancy losses before 20 weeks, and experienced some neonatal deaths (Hernandez et al., 2023), indicating maternal and fetal harms. However, no additional evidence is available on whether the more adverse outcome associated with preconception SARS-CoV-2 infection are part of long COVID.

### 6.6 The endocrine and metabolic system

The induction or aggravation of diabetes maybe the main long-term effect of SARS-CoV-2 on endocrine and metabolic systems (Sherif et al., 2023). On the one hand, SARS-CoV-2 can directly infect the metabolic related tissues (Wu et al., 2021a; Szlachcic et al., 2022; Khreefa et al., 2023). While ACE2 and TMPRSS2 are only modestly expressed in β cells, other SARS-CoV-2 entry factors such as NRP1 and furin exhibit high expression in pancreatic islets, which may potentially explain the tropism of SARS-CoV-2 for β cells (Wu et al., 2021a). The JNK-MAPK pathway is activated after SARS-CoV-2 binding to its receptors, resulting in β cell apoptosis and a subsequent decrease in insulin production and secretion (Wu et al., 2021a). In addition, SARS-CoV-2 could infect and replicate in hepatocytes, leading to a PEPCK-dependent gluconeogenic effect and eventually hyperglycemia in infected patients (Barreto et al., 2023). A glucoregulatory hormone screen revealed a decrease in adiponectin in COVID-19 patients, suggesting that SARS-CoV-2 may invade adipocytes and induce dysfunction in adipose tissue, resulting in insulin resistance (Reiterer et al., 2021). On the other hand, COVID-19 has the potential to induce a similar inflammatory response as observed in diabetes. Infiltration of immune cells in both the exocrine and endocrine pancreas has been observed in COVID-19 patients (Steenblock et al., 2021), indicating a potential impairment of inflammation on β-cell function. Moreover, the proinflammatory environment triggered by a cytokine storm, in which IL-6 plays a primary but not exclusive role, may lead to the development of insulin resistance and dysfunction of β cells (Montefusco et al., 2021; Majidpoor and Mortezaee, 2022).

### 6.7 Extracellular vesicles in long COVID

COVID-19 is an acute disease caused by the infection of SARS-CoV-2, but the symptoms and pathological changes of long COVID persist for months or even years (Bowe et al., 2023). Therefore, one of key questions about long COVID is what are the underlying pathological factors leading to long-term sequelae? Extracellular vesicles (EVs) are vesicles released by donor cells and play an important role in communication by transfer of bioactive molecules between cells and tissues (Xu et al., 2020, Xu et al, 2020; Cheng and Hill, 2022; Liu et al., 2022, Liu et al, 2022b). Growing evidence suggests the key roles of EVs, including circulating levels of EVs and its cargo in disease pathogenesis, potential utility for the development of biomarkers, treatment options, and in vaccination and immunity, and thus the potential involvement of EVs in long COVID is an attractive area of discussion (Nair et al., 2023).

SARS-CoV-2 can be packaged into EVs and released by virus-induced apoptotic cells. In this way, SARS-CoV-2 can achieve immune evasion and universal delivery that bypasses specific receptor-mediated viral entry (Xia et al., 2023). The capacity of EVs to sustain viral infection and facilitate viral establishment in chronic latent infections has been documented (Raab-Traub and Dittmer, 2017). However, there is no direct evidence to prove the persistent infection of SARS-CoV-2 mediated by EVs (Phetsouphanh et al., 2022; Nair et al., 2023), so the role of EVs in long COVID may be more in the regulation of immunity, inflammation and metabolism (**Table 2**) (Nair et al., 2023). Although the current evidence linking EVs and long COVID is extremely limited, numerous pathological factors have been identified in EVs from patients with long COVID, indicating the predictive and diagnostic value of EVs for long COVID.

**Table 2:** Extracellular vesicles in long COVID.

Population	Differences in EVs	Potential effects	Reference
COVID-19 patients after 3 months of recovery	Proteins in lipid metabolic process	Lipid metabolism	(Mao et al., 2021)
	Complement system proteins, Cytokines	Inflammation	
	Cytokines, Antibodies	Immune response	
	Fibrinogen components	Coagulation activity	
	Adipose cell cytokines	Organ dysfunction	
The long‐term impact of infection with novel coronavirus (LIINC) COVID‐19 recovery cohort	Viral proteins and RNAs	Antigen persistence, Over-activate the microglial cells	(Peluso et al., 2022; El-Maradny et al., 2023)
	Mitochondrial proteins	Neuropsychiatric abnormalities	
Patients with long COVID at 1 to 3 months post-infection	Protein markers of neuronal dysfunction	Neurodegeneration	(Sun et al., 2021a; El-Maradny et al., 2023)
Patients at convalescent stages of COVID-19	Lipidomics	Cholesterol metabolism, Immune response, Coagulation processes	(Lam et al., 2021)
Convalescent individuals of COVID-19	Cellular origin and protein content	Prolonged T cell activation	(George et al., 2023)

## 7 Treatments targeting host factors

**Figure 7:**
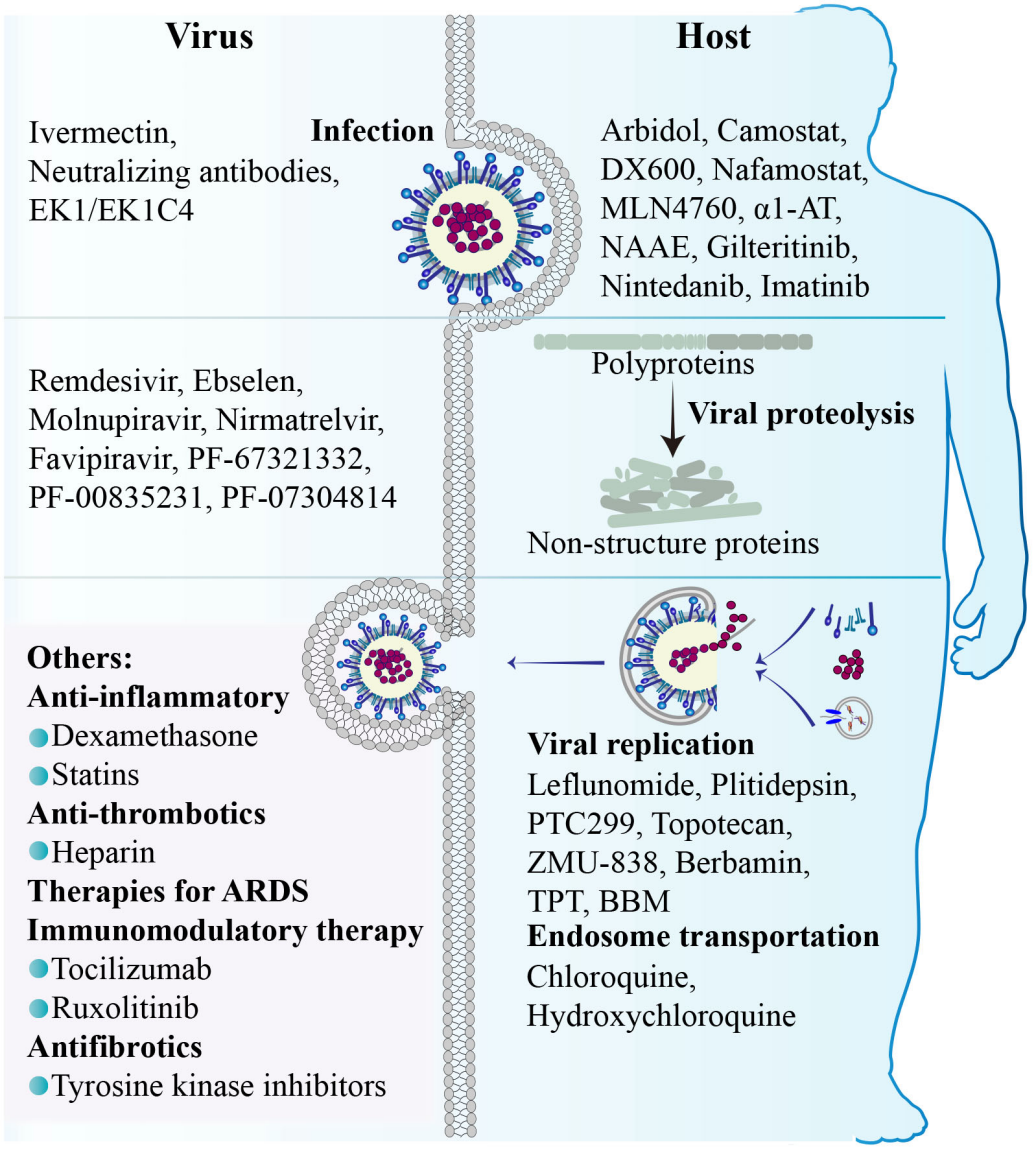
Drugs/Agents for COVID-19. Medicines for COVID-19 are primarily categorized into antivirals, anti-inflammatory drugs, anti-thrombotic medications, acute hypoxemic respiratory failure therapy, immunomodulatory treatment, and anti-fibrosis drugs. The antivirals can be divided into virus-targeted, which primarily act on viral glycoproteins and proteases, and host-targeted, which mainly intervene in the process of virus entry, replication, and transport.

The COVID-19 pandemic has put immense pressure on the global research and clinical communities to develop effective therapeutics. Agents investigated during the pandemic exhibit diverse mechanisms of action: antivirals (e.g. remdesivir, favipiravir), anti-inflammatory (e.g. dexamethasone, statins), anti-thrombotics (e.g. heparin), therapies for acute hypoxaemic respiratory failure, immunomodulatory therapies (e.g. tocilizumab, ruxolitinib), and antifibrotics (e.g. tyrosine kinase inhibitors) (Murakami et al., 2023). Among them, antivirals are regarded as the primary focus of drug development due to their evidence-based effectiveness and significant benefits (**Figure 7**).

Classical antiviral drugs target viral RNAs or proteins, and play a role in specific stages of the SARS-CoV-2 replication cycle, such as entry, proteolytic processing, RNA and protein synthesis, and assembly. For example, Ivermectin blocks the virus-membrane fusion by competitively inhibiting the interaction between the S protein and ACE2 (Yan et al., 2022). Neutralizing antibodies, such as monoclonal antibodies, single-domain antibodies, convalescent plasma and polyclonal antibody preparations, inhibit viral entry into host cells by binding to the S protein and activate host effector pathways to eliminate virus-infected cells (Chen et al., 2023d). The virus-cell fusion can be inhibited by peptides such as EK1, which mimic the SARS-CoV-2 spike HR2 and disrupt the interaction between HR1 and HR2. EK1C4 is a new lipopeptide created based on SARS-CoV-2 structural studies, and can inhibit SARS-CoV-2 fusion more potently than EK1 (Zhou et al., 2022). Antivirals that interfere with the proteolytic processing mainly act on RNA-dependent RNA polymerases (RdRps) and protease enzymes (Tao et al., 2021). The most prevalent antiviral compounds are nucleoside analog polymerase inhibitors, such as Remdesivir, Molnupiravir, and Favipiravir (Jayk Bernal et al., 2022; Amstutz et al., 2023; Shah et al., 2023). Coronaviruses contain two protease enzymes: main protease (Mpro) and papain-like serine protease (PLpro). The investigational Mpro inhibitor PF-00835231 is one of the most potent compounds in its class, its intravenously administered phosphate prodrug (PF-07304814) and oral prodrug (PF-07321332) are being studied in clinical trials (Boras et al., 2021). Nirmatrelvir is an orally Mpro inhibitor with substantial efficacy in patients infected with the delta variant of SARS-CoV-2 (Arbel et al., 2022). Ebselen, an investigational synthetic organoselenium drug, has potential inhibition of Mpro and PLpro (Weglarz-Tomczak et al., 2021). Currently, Ebselen is being evaluated in two clinical trials involving patients with mild-to-moderate and severe COVID-19. While these antivirals may exhibit high selectivity when their targets lack human homologues, they also carry the potential risk of drug resistance due to emerging variants.

In addition to classical antivirals, the strategy of antiviral therapy focusing on host factors interacting with SARS-CoV-2 or acting within pathological pathways offers a potential alternative approach (Lim, 2023). Host-targeted antivirals, as their name suggests, act primarily on host factors involved in the different processes of viral replication cycle. Some compounds exert antiviral effects by acting on host receptors. Arbidol (ARB), as a broad-spectrum antiviral, can interfere with virus entry or the membrane fusion of SARS-CoV-2 (Kang et al., 2023). On the one hand, ARB inhibits the interaction between the S protein and ACE2. On the other hand, it facilitates the internalization of ACE2, leading to a reduction in its cell surface density and subsequently decreasing viral endocytosis (Touret et al., 2020). Some other inhibitors, such as DX600, MLN4760 and NAAE also target ACE2 and effectively inhibit the entry of the virus (Yan et al., 2022). In addition, there are inhibitors that target other receptors of SARS-CoV-2. For instance, gilteritinib and nintedanib specifically target the AXL receptor, while imatinib targets AXL-related proteins (Chen et al., 2023c). Host protease inhibitors also play a crucial role in host-targeted antiviral therapy. Camostat and Nafamostat, both serine protease inhibitors, specifically target TMPRSS2 to effectively inhibit S protein hydrolysis and impede membrane fusion (Fraser et al., 2022). α1-antitrypsin (α1-AT) is an endogenous protease inhibitor that effectively inhibits the protease activity of TMPRSS2. It has reported that patients with α1-AT deficiency (AATD) have a higher risk of developing severe COVID-19 (Rodríguez Hermosa et al., 2023). Host nucleotide and protein synthesis inhibitors have the ability to impede virus replication. Leflunomide, an inhibitor of dihydroorotate dehydrogenase (DHODH), restricts the availability of nucleoside triphosphates necessary for viral replication. Its active metabolite exhibits limited inhibitory activity against SARS-CoV-2 (Xiong et al., 2020). PTC299 and IMU-838 are both investigational DHODH inhibitors currently being evaluated in clinical trials (Hahn et al., 2020; Luban et al., 2021). Plitidepsin, a cyclic peptide derived from marine sources, has demonstrated inhibition of SARS-CoV-2 by targeting eukaryotic translation elongation factor eEF1A (White et al., 2021). We and others have demonstrated that RNA G-quadruplex (RG4), a non-canonical RNA secondary structure within guanine-rich sequences, is widely presented in both SARS-CoV-2 and host factors, including ACE2, AXL, furin, and TMPRSS2, and plays an important role in virus pathogenesis (Liu et al., 2020; Qin et al., 2022; Liu et al., 2022a; Tong et al., 2023). Intriguingly, Topotecan (TPT) and Berbamine (BBM) can function as RG4-stabilizing agents, thereby reducing the expression of RG4-containing host factors and SARS-CoV-2 infection (Liu et al., 2022a; Tong et al., 2023). There are also several compounds disrupting endosomal trafficking within cells. Chloroquine analogs specifically accumulate in acidic intracellular organelles like endosomes and interfere with the viral entry into the cytoplasm. Chloroquine and hydroxychloroquine exhibit moderate efficacy against SARS-CoV-2 by inhibiting the endocytic pathway (Savarino et al., 2003; Zhao et al., 2022).

Compared with antivirals acting on virus, antivirals targeting host factors may offer two advantages: (I) broader spectrum antiviral properties; (II) lower risk of generating immune escape strains and drug-resistant mutants (Shi et al., 2023). Additionally, the majority of these antivirals targeting host factors are repurposed pharmaceuticals that have either obtained regulatory approval or are currently being investigated for alternative medical indications, thereby significantly reducing time and economic costs in drug development.

## 8 Conclusions and perspectives

Host factors are indispensable in viral infection and pathogenesis, with the former encompassing the SARS-CoV-2 replication cycle (including entry, transport, replication, assembly, and release), while the latter involving direct cellular toxicity, aberrant immune responses, metabolic interference, and epigenetic modifications. There are inherent limitations in current screening and validation of potential host factors, since a significant number of screens have relied on bioinformatics analysis or structural analysis and a majority of validation results stemming from *in vitro* experiments. However, it’s undeniable that these findings contribute to the advancement of our comprehension of SARS-CoV-2 and aid in our quest for effective treatments against COVID-19. Due to the extensive involvement of these host factors in normal cellular physiological processes, viral infection-induced alterations in host factors and their associated physiological mechanisms and signaling pathways can lead not only to immediate disease, but also to changes in cell fate, such as cellular senescence, carcinogenesis, or apoptosis, with distant or long-term adverse outcomes.

Cellular senescence is currently recognized as one of the cellular responses to viral infection. Infection with SARS-CoV-2 can induce cellular senescence, leading to the manifestation of senescent phenotypes in infected cells, such as increased DNA damage markers, upregulation of cell-cycle inhibitors like p16^INK4a^ and p21^CIP1^, and the induction of a pro-inflammatory, pro-coagulatory, and pro-fibrotic secretory phenotype known as senescence-associated secretory phenotype (SASP) (Schmitt et al., 2023). It is worth noting that an increased burden of senescent cells is a significant characteristic of aging and metabolic disorders (Tian et al., 2018; Tian et al., 2019), which constitutes a core risk factor for severe COVID-19. Pulmonary fibrosis is a serious sequela of patients with severe COVID-19, and it is also a cellular senescence-driven fatal disease. It has shown that senolytics therapy in patients with idiopathic pulmonary fibrosis displayed improved health function (Justice et al., 2019), and the efficacy of senolytic therapy in reducing senescent cells among patients diagnosed with both diabetes mellitus and chronic kidney disease (Hickson et al., 2019). Consequently, it can be hypothesized that therapies targeting senescence could potentially alleviate the severity and reduce sequela of SARS-CoV-2 infection, as well as other viral infectious diseases.

Tumorigenesis is another undesirable outcome of cell fate changes caused by virus infection. The association between several RNA or DNA virus, including human papillomavirus (HPV), human immunodeficiency syndrome virus (HIV), Kaposi’s sarcoma-associated herpesvirus (KSHV), hepatitis C virus (HCV), and hepatitis B virus (HBV), in cancer initiation and progression has been extensively documented (Tavakolian et al., 2024; Zou et al., 2024). In general, the immune system of COVID-19 patients is suppressed, leading to a decreased abundance of immune cells, altered expression of genes related to immunity, and an intense inflammatory response triggered by viral infection. These factors collectively nurture a favorable microenvironment for tumor occurrence and progression (Xiong and Sun, 2023). Additionally, dysregulation of cellular receptors and proteinases, aberrant metabolism of glucose and lipids, and epigenetic alterations, may serve as the instigator and catalyst for tumorigenesis. The confirmation has been made that cancer patients are at an elevated risk of SARS-CoV-2 infection and exhibit a heightened susceptibility to developing severe COVID-19, consequently leading to increased mortality (Alaeddini and Etemad-Moghadam, 2022). A recent large population-based study reported that patients with COVID-19 admitted to the intensive care unit (ICU) had a higher rate of cancer diagnosis during the subsequent 4 to 10 months follow-up period (Dugerdil et al., 2023). Although this study was unable to establish a definitive correlation between SARS-CoV-2 infection and the development of post-infection tumors, an increased risk of subsequent cancer diagnosis following herpes zoster infection (Cotton et al., 2013) raise the possibility between COVID-19 and subsequent tumorigenesis. Therefore, it is imperative to conduct long-term clinical monitoring on individuals who have recovered from COVID-19, especially severe COVID-19, in order to ascertain any potential heightened susceptibility to cancer.

The COVID-19 pandemic is gradually approaching its conclusion, however the repercussions of COVID-19 are far from abating. The research on SARS-CoV-2 remains significant. The history of human infectious diseases demonstrates that coronavirus epidemics are not isolated incidents; there have been three occurrences of highly pathogenic coronavirus pandemics since 2002. Therefore, our research on coronaviruses should not be fragmented. Host factors, as the pivotal determinants of viral infection and pathogenesis, ought to assume a more prominent role as the central axis in virology investigations. Studying the host factors associated with SARS-CoV-2 will not only yield novel targets for antiviral therapy to combat the current disease, but also facilitate preparedness for potential future pandemics.

## Author contributions

YZ: Formal Analysis, Visualization, Writing – original draft, Writing – review & editing. SC: Visualization, Writing – original draft, Writing – review & editing. YT: Conceptualization, Formal Analysis, Writing – review & editing. XF: Conceptualization, Formal Analysis, Funding acquisition, Resources, Supervision, Visualization, Writing – original draft, Writing – review & editing.

## Glossary

**Table:**

α1-AT	α1-antitrypsin
ACE2	Angiotensin-converting enzyme 2
Ang-II	Angiotensin II
ARB	Arbidol
ARDS	Acute respiratory distress syndrome
BBM	Berbamine
BRD2	Bromodomain containing 2
BST-2	Bone marrow stromal antigen 2
CNS	Central nervous system
COPII	Coatmer protein II complex
COVID-19	Coronavirus disease 2019
CTSL	Cathepsin L
DMVs	Double-membrane vesicles
DPP4	Dipeptidyl peptidase 4
E	Envelope protein
ECM	Extracellular matrix
FGF	Fibroblast growth factor
GLUT2	Glucose transporter 2
HCV	Hepatitis C virus
HDL	High-density lipoprotein
HIV	Human immunodeficiency virus
HMGB1	High mobility group box 1 protein
HOPS	Homotypic fusion and protein sorting complex
HPV	Human papillomavirus
IFN	Interferon
ISGs	Interferon-stimulated genes
KSHV	Kaposi’s sarcoma-associated herpesvirus
LLPS	liquid-liquid phase separation
lncRNAs	Long non-coding RNAs
M	Membrane protein
MAPK	Mitogen-activated protein kinase
MAVS	MDA5-mitochondrial antiviral signaling protein
MERS	Middle East respiratory syndrome
MERS-CoV	MERS-related coronavirus
MHC	Major histocompatibility complexes
Mpro	Main protease
MS	Multiple sclerosis
MyD88	Myeloid differentiation primary response gene 88
N	Nucleocapsid protein
NPC1	Niemann-Picker intracellular cholesterol transporter 1
NRP1	Neuropilin-1
NSPs	Non-structural proteins
NTD	N-terminal domain
ORFs	Open reading frames
OS	Oxidative stress
PABP	polyA-binding protein
PAIP1	PABP-interacting protein 1
PAMPs	Pathogen-associated molecular patterns
pFBs	Pathological fibroblasts
PLpro	Papain-like serine protease
PNS	Peripheral nervous system
PPARα	Peroxisome proliferators-activated receptors α
PRRs	Pattern-recognition receptors
RAB5	RAS-associated protein 5
RBD	Receptor binding domain
RBPs	RNA-binding proteins
RdRp	RNA-dependent RNA polymerase
RG4	RNA G-quadruplex
RIG-I	Retinoic acid inducible gene I
RNP	Ribonucleoprotein
RTCs	Replication-transcription complexes
S	Spike protein
SARS	Severe acute respiratory syndrome
SARS-CoV	SARS-related coronavirus
SASP	Senescence-associated secretory phenotype
SCFAs	Short chain fatty acids
sgRNAs	Sub-genomic RNAs
SNARE	Soluble NSF attachment protein receptor complex
SNPs	Single nucleotide polymorphisms
STAT1	Signal transducer and activator of transcription 1
SUD	SARS-unique domain
T2DM	Type 2 diabetes mellitus
TMEM41B	Transmembrane protein 41B
TMPRSS2	Type II
TNF-β	Tumor necrosis factor beta
TPT	Topotecan
UTRs	Untranslated regions
ZNF579	Zinc finger protein 579
